# Observed changes in China’s methane emissions linked to policy drivers

**DOI:** 10.1073/pnas.2202742119

**Published:** 2022-10-03

**Authors:** Yuzhong Zhang, Shuangxi Fang, Jianmeng Chen, Yi Lin, Yuanyuan Chen, Ruosi Liang, Ke Jiang, Robert J. Parker, Hartmut Boesch, Martin Steinbacher, Jian-Xiong Sheng, Xiao Lu, Shaojie Song, Shushi Peng

**Affiliations:** ^a^Key Laboratory of Coastal Environment and Resources of Zhejiang Province, School of Engineering, Westlake University, Hangzhou, Zhejiang 310024, China;; ^b^Institute of Advanced Technology, Westlake Institute for Advanced Study, Hangzhou, Zhejiang 310024, China;; ^c^Zhejiang Carbon Neutral Innovation Institute, Zhejiang University of Technology, Hangzhou, Zhejiang 310014, China;; ^d^Yangtze River Delta R&D Centre, Monitoring & Assessment Center for GHGs & Carbon Neutrality, China Meteorological Administration, Beijing 100081, China;; ^e^Zhejiang University of Science and Technology, Hangzhou, Zhejiang 310023, China;; ^f^Zhejiang University, Hangzhou, Zhejiang 310058, China;; ^g^National Centre for Earth Observation, University of Leicester, Leicester LE1 7RH, United Kingdom;; ^h^Earth Observation Science, School of Physics and Astronomy, University of Leicester, Leicester LE1 7RH, United Kingdom;; ^i^Empa, Swiss Federal Laboratories for Materials Science and Technology, Duebendorf 8600, Switzerland;; ^j^Center for Global Change Science, Massachusetts Institute of Technology, Cambridge, MA 02139, USA;; ^k^School of Atmospheric Sciences, Sun Yat-sen University, Zhuhai, Guangdong 519082, China;; ^l^State Environmental Protection Key Laboratory of Urban Ambient Air Particulate Matter Pollution Prevention and Control, Tianjin Key Laboratory of Urban Transport Emission Research, College of Environmental Science and Engineering, Nankai University, Tianjin 300350, China;; ^m^China Meteorological Administration-Nankai University Cooperative Laboratory for Atmospheric Environment–Health Research, Tianjin 300350, China;; ^n^Sino-French Institute for Earth System Science, College of Urban and Environmental Sciences, Peking University, Beijing 100871, China

**Keywords:** methane emissions, satellite, China

## Abstract

China is set to actively reduce its methane emissions in the coming decade. A comprehensive evaluation of the current situation can provide a reference point for tracking the country’s future progress. Here, using satellite and surface observations, we quantify China’s methane emissions during 2010–2017. Including newly available data from a surface network across China greatly improves our ability to constrain emissions at subnational and sectoral levels. Our results show that recent changes in China’s methane emissions are linked to energy, agricultural, and environmental policies. We find contrasting methane emission trends in different regions attributed to coal mining, reflecting region-dependent responses to China’s energy policy of closing small coal mines (decreases in Southwest) and consolidating large coal mines (increases in North). Coordinated production of coalbed methane and coal in southern Shanxi effectively decreases methane emissions, despite increased coal production there. We also detect unexpected increases from rice cultivation over East and Central China, which is contributed by enhanced rates of crop-residue application, a factor not accounted for in current inventories. Our work identifies policy drivers of recent changes in China’s methane emissions, providing input to formulating methane policy toward its climate goal.

## Significance

China intends to significantly reduce its methane emissions in the 2020s. A better understanding of methane emissions at regional and national levels provides valuable inputs to the formulation of the action plan. Our observation-based analysis reveals complex and even unexpected linkages between recent changes in China’s methane emissions and related policy drivers: China’s energy policy that prioritizes the phase out of small coal mines leads to region-varying responses in coal mining methane emissions, while agricultural and environmental policies aimed at improving crop production and air quality may have contributed to increased methane emissions from rice cultivation. These findings highlight the importance of integrated considerations in designing methane policy to achieve energy, food, health, and climate targets.

Methane is the second-most-important anthropogenic greenhouse gas, responsible for 1.2 W m^−2^ radiative forcing since industrialization through its direct and indirect radiative effects (1). Sustained growth in global atmospheric methane concentration in the last decade, after a period of stabilization in the early 2000s, has posed additional challenges to meeting the Paris Agreement goals besides increasing CO_2_ concentrations (2, 3), underscoring the urgency of controlling anthropogenic methane emissions.

Methane is emitted from a wide range of human activities, including the production of fossil energy (oil, gas, and coal) and food (livestock, rice cultivation, and freshwater aquaculture) and the treatment of municipal waste (landfill and wastewater) (4). China is the largest anthropogenic methane–emitting country in the world, accounting for ∼15% of the global total (5). Compared with other major national economies (e.g., the United States, Russia, and India), China’s mix of anthropogenic methane sources includes greater contributions from coal mining and rice cultivation, though livestock and municipal waste are also substantial.

Studies disagree on the magnitude of recent trends in China’s methane emissions and their sectoral attributions. While some bottom-up studies indicate a zero-to-negative trend in the last decade after rapid increases in the 2000s because coal production peaked around 2012 (6), inversion-based studies generally infer positive trends but with varied magnitudes and sectoral attributions (7–10) (*SI Appendix*, Table S1). For example, based on an inversion of satellite observations, Miller et al. (8) estimated that China’s anthropogenic methane emissions increased by 1.1 Tg a^−2^ between 2010 and 2015 and attributed the increase almost entirely to the coal sector. This result contrasts with a reduction in coal production during the period and implies an increase in the emission factor (methane emissions per unit mass of coal produced) in response to China’s energy policy. Using the same satellite data but a different prior coal emission distribution, Sheng et al. (9) found a positive but much weaker trend (0.4 Tg a^−2^) and suggested that the sustained increase was instead due to emissions from abandoned coal mines and freshwater aquaculture.

Here, we report China’s methane emissions during 2010–2017 inferred from a high-resolution inverse analysis of satellite and surface observations (*SI Appendix*, Fig. S1) and interpret the inferred trends with bottom-up information and modeling to understand underlying drivers. In addition to satellite and surface data that have been assimilated in previous inversions (7–19), we include in our analysis newly available high-quality surface methane measurements from a network of seven sites distributed across major source regions of China maintained by the China Meteorological Administration (CMA) (20) (see *SI Appendix*, Text S1). Inclusion of additional surface data improves our ability to constrain regional methane emissions and characterize their potential interactions with recent energy (e.g., transition from coal to gas), agricultural, and environmental (e.g., straw open-burning ban) policies in China.

## Results

### China Methane Emissions during 2010–2017.

**Fig. 1.**
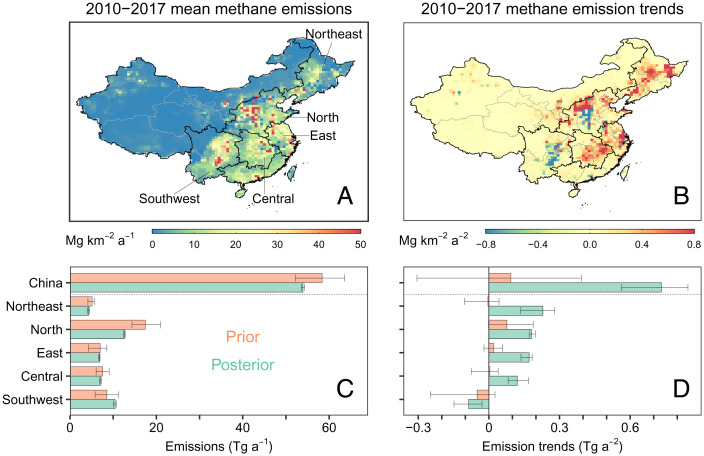
Methane emissions from China inferred by an inversion of satellite and surface observations. Ensemble-averaged posterior estimates are shown for the spatial distribution of 2010–2017 mean methane emissions (*A*) and 2010–2017 linear trends (*B*). Nationally and regionally aggregated prior and posterior estimates are also shown for 2010–2017 mean methane emissions (*C*) and 2010–2017 emission trends (*D*). Five key methane-emitting regions in *C* and *D* are defined spatially in *A*. Error bars in *C* and *D* represent the ranges of the inversion ensemble.

Fig. 1 *A* and *B* show the spatial distribution of ensemble mean posterior methane emissions and their 2010–2017 trends inferred from the inversion of satellite and surface observations. The inversion optimizes methane emissions on spatial groups of varied sizes, with a best resolution of 0.5° × 0.625° (roughly 50 km × 70 km) (*SI Appendix*, Fig. S2; see *Materials and Methods*). Fig. 1 *C* and *D* aggregates these results over major regions (northeast, north, east, central, and southwest) and over the whole of China and compare with prior estimates. *SI Appendix*, Table S2 presents results by province. Our ensemble average posterior estimate for total methane emissions from China is 54 Tg a^−1^ (Fig. 1*C*), which is within the range of bottom-up inventories used as prior information for our inversions (52–64 Tg a^−1^). Subtracting minor contributions from natural sources, our best estimate for the national total anthropogenic emissions is 50 Tg a^−1^, close to the official report to the United Nations Framework Convention on Climate Change (54 Tg a^−1^ for 2014) and is within the range of previous satellite-based inversions (43–59 Tg a^−1^) (8–12, 15–17) (*SI Appendix*, Table S1).

We estimate a linear trend of 0.73 Tg a^−2^ (ensemble range 0.56–0.85 Tg a^−2^) for methane emissions from China during 2010–2017. Positive emission trends in China, ranging from 0.1 to 1.7 Tg a^−2^, were reported in previous inversions for overlapping periods but using only Greenhouse Gases Observing Satellite (GOSAT) observations (8–14) (*SI Appendix*, Table S1). Moreover, our inversion resolves divergent 2010–2017 emission trends at the regional level: positive in major source regions, including Northeast (0.23 Tg a^−2^), North (0.18 Tg a^−2^), East (0.17 Tg a^−2^), and Central China (0.12 Tg a^−2^), but negative in Southwest China (−0.09 Tg a^−2^) (Fig. 1*D*).

Our joint inversion of satellite and surface data infers a smaller national total emission but a larger 2010–2017 emission trend compared with that using only satellite data (total emission: 59 Tg a^−1^; trend: 0.16 Tg a^−2^), driven mainly by differences in Northeast and East China (*SI Appendix*, Figs. S3 and S4). These differences reflect additional information gained from surface observations that improves our capability to constrain methane emissions (*SI Appendix*, Figs. S3–S5; also see *SI Appendix*, Text S2). These surface observations are most valuable for Northeast and East China, where satellite observations are sparse because of frequent cloud and snow conditions. Inclusion of surface measurements in the inversion doubles the degrees of freedom for signals (a measure of observational constraints) for the interannual variabilities of methane emissions from Northeast and East China (*SI Appendix*, Fig. S3), lending more confidence in inferred regional emission trends. Further analysis shows that the improved constraints are mainly provided by Longfengshan (LFS) in Heilongjiang for northeast and Lin'an (LAN) in Zhejiang and Jinsha (JSA) in Hubei for east (*SI Appendix*, Figs. S6 and S7).

**Fig. 2.**
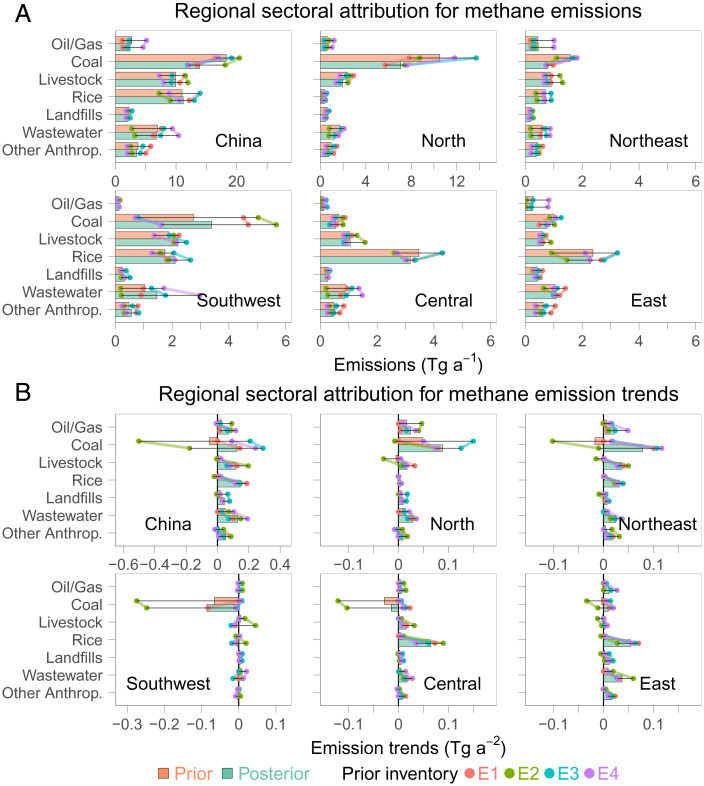
Prior and posterior sectoral methane emissions aggregated nationally and regionally. (*A*) 2010–2017 mean methane emissions; (*B*) 2010–2017 methane emission trends. Bars show ensemble averages, and dots show results for individual members using varied prior inventories.

**Fig. 3.**
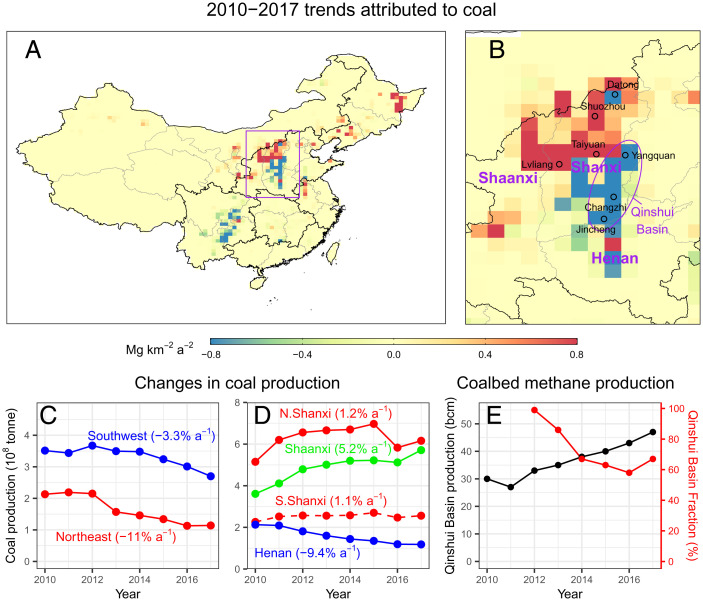
2010–2017 methane emission trends attributed to coal production in China. (*A*) Spatial distribution of coal methane emission trends inferred by the inversion. (*B*) Same as *A* but zoomed in to coal-producing areas in North China (purple box in *A*). (*C*) 2010–2017 coal production in Southwest and Northeast China (21). (*D*) 2010–2017 coal production in northern and southern Shanxi and Henan provinces (21, 22). (*E*) 2010–2017 coalbed methane production from the Qinshui Basin of Shanxi (in billion cubic meters [bcm]) and its fractions relative to national production (22). Cities in Shanxi are denoted in *B* with the purple ellipse showing roughly the Qinshui Basin. Values of regional relative trends in coal production are shown in parentheses in *C* and *D*.

**Fig. 4.**
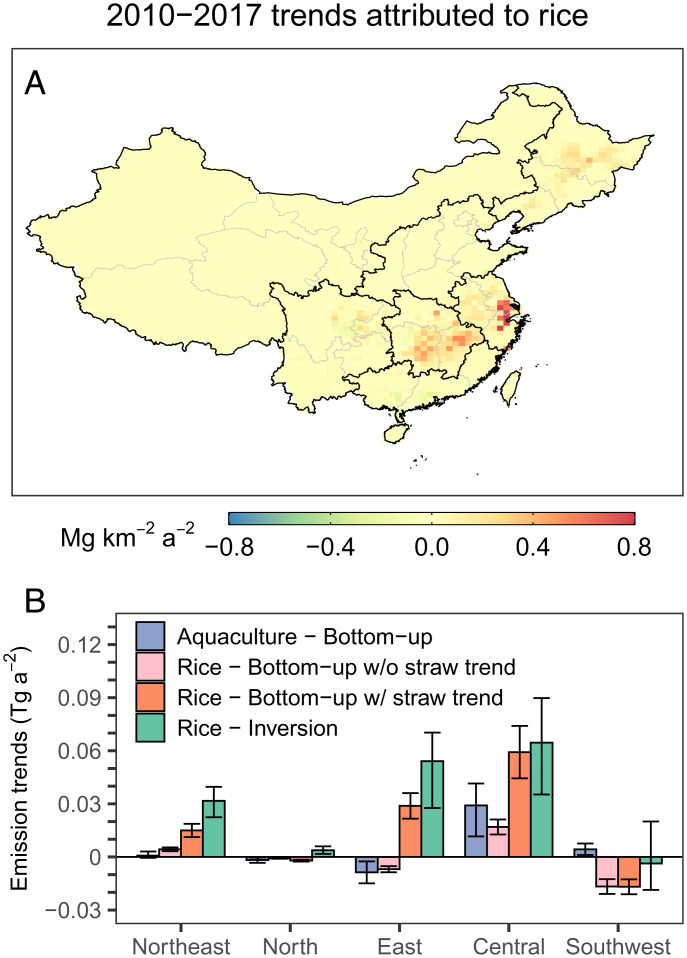
2010–2017 methane emission trends attributed to rice cultivation in China. (*A*) Spatial distribution of rice methane emission trends inferred by the inversion. (*B*) Bottom-up estimates of emission trends from freshwater aquaculture and rice cultivation. Rice emissions are estimated with and without accounting for regional changes in straw retaining. Bottom-up emission trends are compared with inversion inferred trends.

To interpret inferred methane emissions and emission trends, we attribute the inversion results to emission sectors based on their prior fractions in individual grid cells (see *Materials and Methods*). The ensemble ranges (uncertainties) of sectoral emissions (Fig. 2) are notably larger than the total emissions (Fig. 1 *C* and *D*) at national and regional levels, reflecting additional uncertainty from sector attribution. Nevertheless, a few consistent sectoral patterns (i.e., coal and rice) emerge from the ensemble (Figs. 3 and 4), providing insights into underlying drivers for recent changes in China’s methane emissions. In the following sections, we will focus on interpreting 2010–2017 emission trends for coal mining and rice cultivation, two of the most important sectors (accounting for about half of total anthropogenic emissions; Fig. 2*A*) in China.

### Spatially Contrasting Trends in Coal Mine Emissions.

Fig. 3*A* maps the 2010–2017 methane emission trends attributed to the coal sector over China, which shows contrasting patterns across China and even within a smaller geographical region. In North China, which accounts for about 70% of the country’s coal production, we find positive trends (0.14 Tg a^−2^) over Shaanxi, Inner Mongolia, and northern Shanxi and negative trends (−0.05 Tg a^−2^) over Henan and southern Shanxi (Fig. 3*B*). These inferred trends in methane emissions are largely consistent with recent changes in local coal production and reflect China’s energy policy of “consolidation to large coal mines” and “phase out of small coal mines”. For example, despite leveling off or even decline at the national level, production in coal-rich provinces (i.e., Shaanxi, Inner Mongolia, and Shanxi), where most of large coal mines are operated, maintains a steady growth during 2010–2017 (Fig. 3*D*) (21). In contrast, coal production in neighboring Henan province has decreased during the period (Fig. 3*D*) (21), which agrees with observed decreases in methane emissions there.

An exception is in southern Shanxi, where decreasing methane emissions are accompanied by increasing local coal production during 2010–2017 (Fig. 3 *B* and *D*) (22). The area in southern Shanxi with pronounced negative-emission trends coincides with the Qinshui Basin (which encompasses cities of Jincheng, Yangquan, and Changzhi) (Fig. 3*B*), the largest coalbed methane (CBM)-producing basin in China (>60% of national production) (Fig. 3*E*) (22). We suggest that the decrease in methane emissions over the basin is associated with extraction of CBM. The strategy of “extracting coalbed methane before coal mining” adopted in CBM production may lead to sizeable reduction in methane emissions during the coal-mining phase (23). Our inversion results provide observation-based evidence that this strategy practiced in southern Shanxi has been effective in increasing energy supply (both coal and CBM) while at the same time reducing methane emissions.

The inversion also infers a negative emission trend over Southwest China (−0.1 Tg a^−2^) attributed to coal emissions. This decrease in methane emissions is concurrent with a decrease in coal production in this region (Fig. 3*C*), driven by China’s policy of preferentially closing small coal mines with low production output, which are commonly found in Southwest China. Compared with other regions, these coal mines in southwest also have relatively high methane emission factors (methane emissions per unit coal production) (6, 24) (*SI Appendix*, Fig. S8). Therefore, their termination, though driven by energy policy, is also effective from the perspective of methane emission reduction.

Overall, the spatially contrasting trends in coal-methane emissions discussed above are consistent with bottom-up information based on local-level production and emission-factor data. A notable outlier is the northeast, where the inferred positive trend is inconsistent with rapidly declining coal production (Figs. 2 *B* and 3 *C*). Gao et al. (25) suggest a substantial increase in emissions from abandoned coal mines in the northeast, but direct evidence is scarce for now. Aggregating nationally, we find an emission trend of 0.12 Tg a^−2^ (ensemble range −0.2–0.3 Tg a^−2^) from the coal sector during 2010–2017 (Fig. 2*B*). Despite a large uncertainty (ensemble range) due to prior distributions, this estimate is substantially lower than a previous estimate (1.0 Tg a^−2^) by Miller et al. (8) for an overlapping period of 2010–2015 and is more consistent with a lack of coal trend by Sheng et al. (9). This result also indicates a stable national coal-methane emission factor over the 2010–2017 period (*SI Appendix*, Fig. S8). Our analysis demonstrates a complex response of coal-mine emissions to China’s coal policy at regional levels.

### Unexpected Increase in Rice-Cultivation Regions.

Another pattern revealed by our inversion is the positive methane emission trends over East, Central, and Northeast China (Fig. 1*B*). The inversion attributes a large fraction of these trends to rice cultivation (0.14 [0.11–0.19] Tg a^−2^ for China, with 0.03 [0.02–0.04] Tg a^−2^ from Northeast, 0.05 [0.03–0.07] Tg a^−2^ from East, and 0.06 [0.04–0.09] Tg a^−2^ from Central China; brackets show ensemble ranges) (Figs. 2*B* and 4*A*). However, they are not predicted by any of the bottom-up rice-emission inventories used in our inversion ensemble (Fig. 2*B*). This is because interannual trends in most of these bottom-up inventories (E2–E4) are computed based primarily on annual rice cultivation areas (E1 specifies a zero-rice trend), which have changed little or even declined in the last decade (*SI Appendix*, Fig. S9), except for the northeast, where rice areas have increased by 3% a^−1^ (26). Indeed, our own bottom-up calculation (*SI Appendix*, Text S3) also indicates that these observed trends in East and Central China cannot be explained by small changes in rice-cultivation areas (Fig. 4*B*).

Here, we examine two hypotheses. First, we consider that emissions from freshwater aquaculture are not accounted for in our prior bottom-up inventories, but its spatial distribution may well overlap with that of rice paddies (9, 27). To examine this hypothesis, we compute methane emissions from freshwater aquaculture based on provincial data of freshwater aquaculture areas (including pond, river, reservoir, and the rice–fish system, *SI Appendix*, Fig. S9) (*SI Appendix*, Text S4) (28). Using reported emission factors (27), we estimate a national emission of 3.0 Tg a^−1^ (48% of which comes from East and Central China) and a positive but small emission trend of 0.02 Tg a^−2^ (mainly from Central China) (Fig. 4B). This calculation suggests that freshwater aquaculture, though an important missing sector in current bottom-up inventories, is unlikely to explain the inferred positive trends over rice-cultivation regions (0.14 Tg a^−2^).

We then consider that, in addition to cultivation areas, methane emissions from rice paddies are also affected by management practices, for instance, the amount of organic fertilizer applied. These factors are assumed constant in our prior inventories. During the last decade, many regions in China have seen an increasing rate of straw-residue application (29, 30) (*SI Appendix*, Fig. S10), which is promoted by China’s agricultural policy to increase use of organic fertilizers to maintain soil organic carbon and boost crop production (31). Meanwhile, this increase in straw-residue application is also driven by increasingly strict bans on crop-residue burning in recent years for air-pollution control (32). However, its potential side effect, enhanced methane emissions from rice paddies (33, 34), remains unevaluated. We evaluate this effect by recomputing 2010–2017 rice-methane emissions following Yan et al. (35) but now accounting for regional changes in straw retaining fractions (*SI Appendix*, Text S3). We find positive rice-emission trends of 0.1 Tg a^−2^ in major rice-cultivation regions (0.015 Tg a^−2^ for Northeast China, 0.06 Tg a^−2^ for Central China, and 0.03 Tg a^−2^ for East China), which can explain appreciable fractions of trends inferred by the inversion (0.14 Tg a^−2^) (Fig. 4B). This result showcases the interactive impacts of agricultural, environmental, and climate policies and underscores the demand for improved management and technology for crop production, air pollution, and greenhouse-gas mitigation (36, 37).

## Discussion

Our inversion of atmospheric-methane observations infers an increase of China’s methane emissions at a rate of 0.73 Tg a^−2^ during 2010–2017, indicating a slowdown from a higher rate inferred for the pre-2010 period (1.1–1.2 Tg a^−2^) (17, 19), though observational data were even sparser then. We show that the 2010–2017 increase is attributable to multiple emission sectors (Fig. 2B) instead of primarily to the coal sector, as suggested previously (8). Our best estimate of the national emission trend from coal mining is relatively small (0.12 Tg a^−2^), consistent with a stabilization of China’s coal production during 2010–2017. We also find increases in methane emissions attributable to rice cultivation (0.14 Tg a^−2^), which is inconsistent with current bottom-up estimates but can be explained with changes in agricultural practices. In addition to the coal and rice sectors, which have been the focus of our discussion, other sectors also contribute to the positive national emission trend. For example, we find an increase of 0.12 Tg a^−2^ in livestock emissions driven mainly by Northeast China. The inversion also attributes a positive trend of 0.2 Tg a^−2^ to waste treatment (wastewater and landfills), which is partly explained by the increasing volume of wastewater and solid waste in China (6). However, it is possible that a rapid increase of natural-gas use in Chinese cities, which spatially overlaps with waste treatment, is also a nonnegligible contributor. Our inversion does not evaluate this source, because it is not included in current bottom-up inventories due to a lack of information. We suggest that further investigations are necessary to assess their contributions to urban methane emissions in China.

Based on our inversion results, we have evaluated the impact of China’s policies on methane emissions from two major methane-emitting sectors, coal mining and rice cultivation. Our analysis reveals spatially contrasting coal-methane emission trends at the regional level, reflecting complex region-dependent responses to China’s energy policy that prioritizes closing small coal mines (e.g., southwest) and consolidating large coal mines (e.g., north). Our results also provide evidence that coordinated production of CBM and coal (extracting CBM before coal mining) (southern Shanxi), encouraged by the current policy, is effective in reducing methane emissions while maintaining energy supply. In addition, we detect increasing methane emissions over rice-cultivation regions (e.g., East and Central China), unexpected from rice-cultivation area and production data. Bottom-up calculations suggest that this increase is likely a side effect from increasing rates in crop-residue incorporation, a practice promoted by current policies targeted at improving air quality and raising crop yields. Although this effect is well known in field studies, our evaluation shows that it can drive emission trends at regional and national scales. Our findings demonstrate the interplay between methane emissions and policy drivers on a subnational level, providing information that can support the formulation of China’s action plan on reducing methane emissions.

## Materials and Methods

### Methane Observations.

We use surface methane observations from seven sites operated by the CMA (20, 38) and six sites from the World Data Centre for Greenhouse Gases (WDCGG) of World Meteorological Organization's Global Atmosphere Watch program. Locations of these sites are shown in *SI Appendix*, Fig. S1. The CMA sites are distributed in different regions of China, while the WDCGG sites are in regions neighboring mainland China. Methane mole fractions are measured weekly with discrete flask air samples at five surface sites (two CMA and three WDCGG) and hourly with continuous in situ observation at the other eight sites (five CMA and three WDCGG). CMA measurements that are influenced by very local emissions are filtered out following a protocol developed in Fang et al. (20). For hourly measurements, daily daytime averages are used for our inversion. See *SI Appendix*, Text S1 and Table S3 for more information on the surface measurements.

We also use satellite column methane data collected by the thermal and near infrared sensor for carbon observation-Fourier transform spectrometer (TANSO-FTS) instrument on board the GOSAT. The nadir-viewing instrument, launched in 2009, measures methane in the 1.65-μm band from a sun-synchronous orbit with a local overpass time around 13:00 (39). We use the University of Leicester version 9 CO_2_ proxy retrieval (40), which contains in total ∼400,000 successful retrievals over land within our inversion domain for 2010–2017 (*SI Appendix*, Fig. S1). Evaluation against ground-based network showed that the retrieval has a single-observation precision of 13.7 parts per billion by volume (ppbv) and a regional bias of 4 ppbv (40).

*SI Appendix*, Text S5 describes the method to specify errors for these surface and satellite observations, and *SI Appendix*, Table S4 summarizes error statistics (variance and correlation scales) used in our inversion.

### Model Simulations.

We use the nested version of the GEOS-Chem chemical transport model (12.9.3) to describe the relationship between surface-methane emissions and atmospheric-methane concentrations (41, 42). The model simulation is conducted at 0.5° × 0.625° resolution in the East Asia domain (15°–55°N, 60°–145°E) and is driven by Modern-Era Retrospective Analysis for Research and Applications, version 2 (MERRA-2) reanalysis meteorological fields from the NASA Global Modeling and Assimilation Office (43). To simulate atmospheric methane, we use a set of methane emission inventories that provide spatially resolved surface-methane fluxes for a variety of anthropogenic and natural sources. We use 3-h dynamic boundary conditions (i.e., methane concentrations at the lateral edges of the modeling domain) from a global 4° × 5° inversion of satellite (GOSAT) (44) and in situ (GLOBALVIEWplus CH_4_ ObsPack) (45) observations by Lu et al. (12). The atmospheric oxidation of methane in our regional simulation is computed using a monthly OH field from a full chemistry GEOS-Chem simulation. We sample methane fields simulated by GEOS-Chem at the location and time of satellite or surface observations. To compare with surface observations, we compute simulated daily daytime averages following the exact procedure applied to hourly surface observations. To compare with satellite observations, we compute column-averaged methane mole fractions by accounting for the vertical sensitivity of the instrument and the prior vertical profile of methane. We also correct the known GEOS-Chem model biases in the stratosphere following Zhang et al. (11), though these biases are small for midlatitude conditions.

### Bottom-Up Emission Inventories.

We compile a bottom-up emission inventory (E1) to drive our methane simulations. For anthropogenic emissions, we use the global inventory Emissions Database for Global Atmospheric Research (EDGAR) v4.3.2 for 2012 (46), with its emissions from China’s coal production replaced by a regional inventory with improved location information for coal mines (24). For natural-methane emissions, we consider wetlands from WetCHARTs (47), biomass burning from the Global Fire Emissions Database (GFED4s) (48), geological seeps by Maasakkers et al. (7), and termites by Fung et al. (49). These inventories yield a total methane emission of 62 Tg a^−1^ from China, with 58 Tg a^−1^ from anthropogenic sources and 4 Tg a^−1^ from natural sources (Fig. 2*A*). In our inversion, we use these emission inventories as prior estimates and optimize them to best fit the observations.

To characterize the inversion sensitivity to prior choices, we use a suite of alternative bottom-up estimates, including PKU-CH_4_ v2 (E2) (6), EDGAR v5.0 (E3) (50), and CEDS-2021–04-21 (E4) (51), as prior information for anthropogenic methane emissions (Fig. 2*A* and *SI Appendix*, Table S5). These inventories differ in their spatial, temporal, and sectoral distributions. For instance, PKU-CH_4_ v2 predicts pronounced decreases in coal emissions from many regions of China, driven by coal-production data being used, as compared with other inventories that find either close-to-zero or positive trends. Natural emissions are not perturbed. We present averages and ranges of this inversion ensemble in this study.

To explore factors driving inferred increases in methane emissions over rice-cultivation regions, we conduct additional bottom-up emission modeling for freshwater aquaculture and rice cultivation, based on annual province-level data from statistical yearbooks (freshwater aquaculture areas from China Fishery Statistical Yearbook (28) and rice-cultivation areas from China Agriculture Yearbook (26)).We take emission factors for freshwater aquaculture from Yuan et al. (27) and those for rice cultivation (as a function of region, rice season, water regime, and organic input) from Yan et al. (35). See *SI Appendix*, Texts S3 and S4 for more information.

### Observation-based Emission Estimations.

We apply observations from satellite and surface networks (observation vector ***y***) to optimize methane emissions from East Asia for the period of 2010–2017. The optimized methane emission flux (${\hat{E}}_{\mathrm{gys}}$) at location *g*, year *y*, and season *s* is given by

[1] $$\begin{array}{c} {\hat{E}}_{\mathrm{gys}}=\left(a_{g}+b_{gy}+c_{gs}\right)E_{\mathrm{gys}}^{a}, \end{array}$$

where $E_{\mathrm{gys}}^{a}$ is the prior emission estimate. $a_{g}$, $b_{gy}$, and $c_{gs}$ represent unitless scaling coefficients for the 2010–2017 averages, annual anomalies, and seasonal anomalies, respectively. *g* represents index for 600 spatial groups (*SI Appendix*, Fig. S2), aggregated from the original 0.5° × 0.625° model grid, in order to preserve high resolution for regions with strong or concentrated emissions, while reducing resolution (and hence computation) in regions with weak or diffuse emissions (52). *y* represents the year between 2010 and 2017 (8 y), and *s* represents a season in January/February/March, April/May/June, July/August/September, or October/November/December (four seasons). We assemble the scaling coefficients $\;a_{g}$, $b_{gy}$, and $c_{gs}$ in a state vector ***x*** containing, in total, 600 × (1 + 8 + 4) = 7,800 elements.

We then solve optimal posterior estimates ($\hat{x}$) in the Bayesian framework assuming normal errors:

[2] $$\begin{array}{c} \hat{x}=x_{\text{a}}+{\left(K^{\text{T}}S_{\text{O}}^{-1}K+S_{\text{a}}^{-1}\right)}^{-1}K^{\text{T}}S_{\text{O}}^{-1}\left(y-Kx_{\text{a}}\right). \end{array}$$

Here, the Jacobian matrix, **K**, describes the relationship between methane concentrations and emissions (∂***y***/∂***x***) and is constructed explicitly from a series of perturbed model simulations. $x_{\text{a}}$ is the prior estimate for ***x***.** S**_a_ is the prior error covariance matrix that represents error statistics for $x_{\text{a}}$, and **S**_O_ is the observation error covariance matrix that includes both the instrument error and the forward model error. We take **S**_a_ as a diagonal matrix and specify 50% uncertainty for emission fluxes. Detailed information on construction of **S**_O_ is in *SI Appendix*, Text S5.

Estimates of sectoral emissions provide additional insight. We update sectoral emission estimates, ${\hat{E}}_{\mathrm{gys}}^{i}$ (*i* denotes an emission sector, which can be oil/gas, coal, livestock, rice, landfills, and wastewater), based on the fraction of the sector *i* in prior emissions at given location (*g*) and time (year *y* and season *s*):

[3] $$\begin{array}{c} {\hat{E}}_{\mathrm{gys}}^{i}=\frac{E_{\mathrm{gys}}^{a,i}}{E_{\mathrm{gys}}^{a}}{\hat{E}}_{\mathrm{gys}} \end{array}.$$

Eq. **3** implicates that, in addition to total fluxes ${\hat{E}}_{\mathrm{gys}}$, the uncertainty of sectoral emissions also arises from the $\frac{E_{\mathrm{gys}}^{a,i}}{E_{\mathrm{gys}}^{a}}$ term, which is inventory dependent and unoptimized by the inversion. To capture this uncertainty, we form an ensemble of inversions by using varied emission inventories as prior information (see *SI Appendix*, Table S5). This is readily done by replacing the prior estimate $x_{\text{a}}$ in Eq. **2**, which incurs little additional computation once the Jacobian matrix **K** is constructed.

Our inversion method gives a closed-form solution to the posterior error covariance matrix ($\hat{S}$), describing the error structure of $\hat{x}$, and the averaging kernel matrix (A), describing the sensitivity of the solution to the true state (∂$\hat{x}$/∂***x***). In addition to perturbing prior choices, we also analyze this information to understand uncertainties of the inversion results (see *SI Appendix*, Text S6 for more discussion). We also perform inversions with only GOSAT satellite observations to compare with our main inversions using both GOSAT and surface data, to evaluate how inclusion of surface observations affects our inferences (see *SI Appendix*, Text S2).

## Supplementary Material

Supplementary File

## Data Availability

The data supporting the findings of this study are available through a public repository [https://doi.org/10.57760/sciencedb.02269,(53)].

## References

[1] S. Szopa , “Climate change 2021: The physical science basis” in Contribution of Working Group I to the Sixth Assessment Report of the Intergovernmental Panel on Climate Change, V. Masson-Delmotte , Eds. (Cambridge University Press, 2021).

[2] E. G. Nisbet , Very strong atmospheric methane growth in the 4 years 2014–2017: Implications for the Paris agreement. Global Biogeochem. Cycles 33, 318–342 (2019).

[3] A. L. Ganesan , Advancing scientific understanding of the global methane budget in support of the Paris agreement. Global Biogeochem. Cycles 33, 1475–1512 (2019).

[4] S. Kirschke , Three decades of global methane sources and sinks. Nat. Geosci. 6, 813 (2013).

[5] UNFCCC, Greenhouse Gas Inventory Data Interface (2021). https://di.unfccc.int/detailed_data_by_party. Accessed 1 December 2021.

[6] G. Liu , Recent slowdown of anthropogenic methane emissions in China driven by stabilized coal production. Environ. Sci. Technol. Lett. 8, 739–746 (2021).

[7] J. D. Maasakkers , Global distribution of methane emissions, emission trends, and OH concentrations and trends inferred from an inversion of GOSAT satellite data for 2010–2015. Atmos. Chem. Phys. 19, 7859–7881 (2019).

[8] S. M. Miller , China’s coal mine methane regulations have not curbed growing emissions. Nat. Commun. 10, 303 (2019). 30696820 10.1038/s41467-018-07891-7 PMC6351523

[9] J. Sheng , Sustained methane emissions from China after 2012 despite declining coal production and rice-cultivated area. Environ. Res. Lett. 16, 104018 (2021).

[10] M. Saunois , The global methane budget 2000–2017. Earth Syst. Sci. Data 12, 1561–1623 (2020).

[11] Y. Zhang , Attribution of the accelerating increase in atmospheric methane during 2010–2018 by inverse analysis of GOSAT observations. Atmos. Chem. Phys. 21, 3643–3666 (2021).

[12] X. Lu , Global methane budget and trend, 2010–2017: Complementarity of inverse analyses using in situ (GLOBALVIEWplus CH4 ObsPack) and satellite (GOSAT) observations. Atmos. Chem. Phys. 21, 4637–4657 (2021).

[13] Y. Yin , Accelerating methane growth rate from 2010 to 2017: Leading contributions from the tropics and East Asia. Atmos. Chem. Phys. 21, 12631–12647 (2021).

[14] F. Wang , Interannual variability on methane emissions in monsoon Asia derived from GOSAT and surface observations. Environ. Res. Lett. 16, 024040 (2021).

[15] F. Wang , Methane emission estimates by the global high-resolution inverse model using national inventories. Remote Sens. 11, 2489 (2019).

[16] R. Janardanan , Country-scale analysis of methane emissions with a high-resolution inverse model using GOSAT and surface observations. Remote Sens. 12, 375 (2020).

[17] A. R. Stavert , Regional trends and drivers of the global methane budget. Glob. Change Biol. 28, 182–200 (2022). 10.1111/gcb.15901 PMC9298116 34553464

[18] P. Bergamaschi , Atmospheric CH4 in the first decade of the 21st century: Inverse modeling analysis using SCIAMACHY satellite retrievals and NOAA surface measurements. J. Geophys. Res. Atmospheres 118, 7350–7369 (2013).

[19] R. L. Thompson , Methane emissions in East Asia for 2000–2011 estimated using an atmospheric Bayesian inversion. J. Geophys. Res. D Atmospheres 120, 4352–4369 (2015).

[20] S.-X. Fang, L.-X. Zhou, K. A. Masarie, L. Xu, C. W. Rella, Study of atmospheric CH4 mole fractions at three WMO/GAW stations in China. J. Geophys. Res. D Atmospheres 118, 4874–4886 (2013).

[21] National Bureau of Statistics of China, China Statistical Yearbook 2010-2018 (China Statistics Press, Beijing, 2018).

[22] *Shanxi Provincial Bureau of Statistics, Survey Office of the National Bureau of Statistics in Shanxi, *Shanxi Statistical Yearbook 2010-2018** (China Statistics Press, Beijing, China, 2018).

[23] S. Tao, S. Chen, Z. Pan, Current status, challenges, and policy suggestions for coalbed methane industry development in China: A review. Energy Sci. Eng. 7, 1059–1074 (2019).

[24] J. Sheng, S. Song, Y. Zhang, R. G. Prinn, G. Janssens-Maenhout, Bottom-up estimates of coal mine methane emissions in China: A gridded inventory, emission factors, and trends. Environ. Sci. Technol. Lett. 6, 473–478 (2019).

[25] J. Gao, C. Guan, B. Zhang, K. Li, Decreasing methane emissions from China’s coal mining with rebounded coal production. Environ. Res. Lett. 16, 124037 (2021).

[26] *Ministry of Agriculture and Rural Affairs of China, *China Agriculture Yearbook 2010-2018** (China Agriculture Press, Beijing, 2018).

[27] J. Yuan , Rapid growth in greenhouse gas emissions from the adoption of industrial-scale aquaculture. Nat. Clim. Chang. 9, 318–322 (2019).

[28] *Fisheries Administration of the Ministry of Agriculture and Rural Affairs of China, *China Fishery Statistical Yearbook 2010-2018** (China Agriculture Press, Beijing, 2018).

[29] Z. Shi , Utilization characteristics, technical model and development suggestion on crop straw in China. J. Agric. Sci. Technol. 21, 8–16 (2019).

[30] G. Zhang , Residue usage and farmers′ recognition and attitude toward residue retention in China′s croplands. Nongye Huanjing Kexue Xuebao 36, 981–988 (2017).

[31] C. Liu, M. Lu, J. Cui, B. Li, C. Fang, Effects of straw carbon input on carbon dynamics in agricultural soils: A meta-analysis. Glob. Change Biol. 20, 1366–1381 (2014). 10.1111/gcb.12517 24395454

[32] L. Huang , Assessment of the effects of straw burning bans in China: Emissions, air quality, and health impacts. Sci. Total Environ. 789, 147935 (2021). 34049144 10.1016/j.scitotenv.2021.147935

[33] P. Hou , Methane emissions from rice fields under continuous straw return in the middle-lower reaches of the Yangtze River. J. Environ. Sci. (China) 25, 1874–1881(2013). 24520731 10.1016/s1001-0742(12)60273-3

[34] Y. Jiang , Acclimation of methane emissions from rice paddy fields to straw addition. Sci. Adv. 5, eaau9038 (2019). 30746466 10.1126/sciadv.aau9038 PMC6357747

[35] X. Yan, Z. Cai, T. Ohara, H. Akimoto, Methane emission from rice fields in mainland China: Amount and seasonal and spatial distribution. J. Geophys. Res. D Atmospheres 108, 4505 (2003).

[36] Y. Cao , Mitigating the global warming potential of rice paddy fields by straw and straw-derived biochar amendments. Geoderma 396, 115081 (2021).

[37] Q. Nan, C. Fang, L. Cheng, W. Hao, W. Wu, Elevation of NO_3_^-^-N from biochar amendment facilitates mitigating paddy CH_4_ emission stably over seven years. Environ. Pollut. 295, 118707 (2022). 34923062 10.1016/j.envpol.2021.118707

[38] J. Wang , Large Chinese land carbon sink estimated from atmospheric carbon dioxide data. Nature 586, 720–723 (2020). 33116288 10.1038/s41586-020-2849-9

[39] A. Kuze, H. Suto, M. Nakajima, T. Hamazaki, Thermal and near infrared sensor for carbon observation Fourier-transform spectrometer on the Greenhouse Gases Observing Satellite for greenhouse gases monitoring. Appl. Opt. 48, 6716–6733 (2009). 20011012 10.1364/AO.48.006716

[40] R. J. Parker , A decade of GOSAT Proxy satellite CH_4_ observations. Earth Syst. Sci. Data 12, 3383–3412 (2020).

[41] J. D. Maasakkers , 2010–2015 North American methane emissions, sectoral contributions, and trends: A high-resolution inversion of GOSAT observations of atmospheric methane. Atmos. Chem. Phys. 21, 4339–4356 (2021).

[42] X. Lu , Methane emissions in the United States, Canada, and Mexico: evaluation of national methane emission inventories and sectoral trends by inverse analysis of in situ (GLOBALVIEWplus CH4 ObsPack) and satellite (GOSAT) atmospheric observations. Atmos. Chem. Phys. 2022, 395–418 (2022).

[43] R. Gelaro , The modern-era retrospective analysis for research and applications, version 2 (MERRA-2). J. Clim. 30, 5419–5454 (2017). 32020988 10.1175/JCLI-D-16-0758.1 PMC6999672

[44] R. J. Parker, H. Boesch, University of Leicester GOSAT Proxy XCH4 v9.0 (Centre for Environmental Data Analysis, 2020).

[45] A. G. Di Sarra , Multi-laboratory compilation of atmospheric carbon dioxide data for the period 1983–2020; obspack_ch4_1_GLOBALVIEWplus_v4.0_2021-10-14. NOAA Global Monitoring Laboratory, 10.25925/20211001 Accessed 15 September 2022..

[46] G. Janssens-Maenhout , EDGAR v4.3.2 Global Atlas of the three major Greenhouse Gas Emissions for the period 1970-2012. Earth Syst. Sci. Data Discuss. 2017, 1–55 (2017).

[47] A. A. Bloom , A global wetland methane emissions and uncertainty dataset for atmospheric chemical transport models (WetCHARTs version 1.0). Geosci. Model Dev. 10, 2141–2156 (2017).

[48] G. R. van der Werf , Global fire emissions estimates during 1997–2016. Earth Syst. Sci. Data 9, 697–720 (2017).

[49] I. Fung , Three-dimensional model synthesis of the global methane cycle. J. Geophys. Res. D Atmospheres 96, 13033–13065 (1991).

[50] M. Crippa , Fossil CO2 and GHG Emissions of All World Countries - 2019 Report, EUR 29849 EN (Publications Office of the European Union, Luxembourg, 2019).

[51] R. M. Hoesly , Historical (1750–2014) anthropogenic emissions of reactive gases and aerosols from the Community Emissions Data System (CEDS). Geosci. Model Dev. 11, 369–408 (2018).

[52] A. J. Turner, D. J. Jacob, Balancing aggregation and smoothing errors in inverse models. Atmos. Chem. Phys. 15, 7039–7048 (2015).

[53] Y. Zhang, S. Fang, 2010-2017 China's methane emissions inferred from an inversion of satellite and surface observations. ScienceDB. https://www.scidb.cn/en/detail?dataSetId=04e7d3c5658d468e833496c713178f89. Deposited 17 August 2022.

