# Supplementary material for: Observed changes in China’s methane emissions linked to policy drivers
Source: Proc Natl Acad Sci U S A. 2022 Oct 3;119(41):e2202742119. doi: 10.1073/pnas.2202742119 (PMC9564322; doi:10.1073/pnas.2202742119)
Supplement: Supplementary File [file pnas.2202742119.sapp.pdf]

Supplementary Materials for

Observed Changes in China's Methane Emissions Linked to Policy Drivers

Yuzhong Zhang[#*1,2], Shuangxi Fang[#*3,4], Jianmeng Chen[*5], Yi Lin[3], Yuanyuan Chen[3], Ruosi Liang[1,2,6], Ke Jiang[1,2], Robert Parker[7,8], Hartmut Boesch[7,8], Martin Steinbacher[9], Jian-Xiong Sheng[10], Xiao Lu[11], Shaojie Song[12,13], Shushi Peng[14]

[1] Key Laboratory of Coastal Environment and Resources of Zhejiang Province, School of Engineering, Westlake University, Hangzhou, Zhejiang, China
[2] Institute of Advanced Technology, Westlake Institute for Advanced Study, Hangzhou, Zhejiang, China
[3] Zhejiang Carbon Neutral Innovation Institute, Zhejiang University of Technology, Hangzhou, Zhejiang, China
[4] Yangtze River Delta R&D Centre, Monitoring & Assessment Center for GHGs & Carbon Neutrality, China Meteorological Administration, Beijing, China
[5] Zhejiang University of Science and Technology, Hangzhou, Zhejiang, China
[6] Zhejiang University, Hangzhou, Zhejiang, China
[7] National Centre for Earth Observation, University of Leicester, Leicester, UK
[8] Earth Observation Science, School of Physics and Astronomy, University of Leicester, Leicester, UK
[9] Empa, Swiss Federal Laboratories for Materials Science and Technology, Duebendorf, Switzerland
[10] Center for Global Change Science, Massachusetts Institute of Technology, Cambridge, MA, USA
[11] School of Atmospheric Sciences, Sun Yat-sen University, Zhuhai, Guangdong, China
[12] State Environmental Protection Key Laboratory of Urban Ambient Air Particulate Matter Pollution Prevention and Control, Tianjin Key Laboratory of Urban Transport Emission Research, College of Environmental Science and Engineering, Nankai University, Tianjin, China
[13] CMA-NKU Cooperative Laboratory for Atmospheric Environment–Health Research, Tianjin, China
[14] Sino-French Institute for Earth System Science, College of Urban and Environmental Sciences, Peking University, Beijing, China

*Correspondence to:

Yuzhong Zhang: zhangyuzhong@westlake.edu.cn
Shuangxi Fang: fangsx@zjut.edu.cn
Jianmeng Chen: jchen@zjut.edu.cn

#Yuzhong Zhang and Shuangxi Fang contribute equally to this work.

Text S1 Surface observations from the CMA network

We use surface methane observations from an observation network of 7 sites maintained by China Meteorological Administration (CMA), under the framework of World Meteorological Organization/Global Atmosphere Watch program (WMO/GAW). We use hourly or weekly CH_4 data from 2010–2017 at seven stations in CMA, including one WMO/GAW global station, three WMO/GAW regional stations, and three CMA background stations. Location of the seven stations is illustrated in Figure S1 and Table S3.

Site Locations

The Waliguan Baseline (WLG) Observatory is a WMO/GAW global atmospheric background station in China and the only global atmospheric background station in inland Eurasia. The observatory is built on the top of mountain Waliguan in the northeast of the Tibet plateau. Greenhouse gases measurements from this site provide essential information on sources and sinks from within the Eurasian continent because of its unique location. The observed data at the site is submitted to World Data Center of Greenhouse Gases (WDCGG) and used for many scientific products (e.g., Global View database, WMO/GAW GHGs bulletin).

The LAN station is located at the center of the Yangtze Delta area, 50 km from Hangzhou and 150 km from Shanghai. The Yangtze Delta is one of the largest economic zones in China. The SDZ station is located 150 km northeast of Beijing and can provide a constraint on fluxes from the North China plain, another important economic zone of China. The LFS station is in the Northeast Plain, 175 km southeast of Harbin. The Northeast Plain is one the most important rice producing regions in China, and is poorly observed by satellite. The XGL station is situated in Southwest China, 30 km north of Shangri-La County and 460 km from Kunming (the capital of Yunnan province). It resides in the transition zone of the Yunnan-Guizhou Plateau and the Tibetan Plateau. The JSA station is in the Jianghan Plain. AKD is in the Xinjiang autonomous region in Northwest China, and provides a constraint on the Gobi wetlands that reside between grassland and sparsely vegetated regions.

Instruments

All stations continuously measure CH_4 mole fractions using cavity ring-down spectroscopy (CRDS) analyzers, except for JSA and AKD where discrete flask sampling is made. The model of instruments was initially G1301 (Picarro Inc. USA) and then was updated to G2401 (Picarro Inc. USA) in 2015. Schematic of the analytical system is described by Fang, et al. [1] and Wang, et al. [2].

Air sample is delivered to the instrument by a vacuum pump (N022, KNF Neuberger, Germany) via a dedicated 10 mm o.d. sampling line (Synflex 1300 tubing, Eaton, USA). Then the ambient air is filtered (7 μm) and pressurized at 1 atm. The ambient air is dried to a dew point of approximately -60 °C by passing it through a glass trap submerged into a -70 °C ethanol bath (MC480D1, SP Industries, USA). An automated sampling module equipped with a VICI 8 port multi-position valve is designed to sample from separate gas streams (standard gas cylinders and ambient air). The residence time of the air from the top of the inlet to the analyzers is less than 60 s. Further details about the measurement strategy are described by Fang, et al. [1].

The CH_4 mole fractions at JSA and AKD are measured as part of a larger discrete flask sampling program in China, which is similar to the NOAA flask program [3]. Two samples are collected in series using glass flasks and a portable battery powered sampling apparatus with a 5 m (agl) intake height. Weekly flask samples are collected at JSA and AKD and shipped to Beijing where they are analyzed for CH_4. The CH_4 in flask samples are analyzed by a Picarro G2301 (Picarro Inc. USA) system with a flow rate of 150 mL min^{-1}. The repeatability of flask-Picarro system is approximately 1 ppb.

Calibration

The in situ CH_4 observation systems are routinely calibrated against the standards propagated by the WMO/GAW Central Calibration Laboratory operated by NOAA/ESRL using the WMO X2004A scale [3]. Methane mole fractions are referenced to a Working High (WH) and a Working Low (WL) standard by using a linear two-point fit through the most recent standard gas measurements (WH & WL). Additionally, a calibrated cylinder filled with compressed ambient air is used as a target gas to check the precision and stability of the system routinely. All standard gases are pressurized in 29.5 L treated aluminum alloy cylinders (Scott-Marrin Inc) fitted with high-purity, two-stage gas regulators. An automated sampling module equipped with a VICI 8 port multi-position valve is designed to sample from separate gas streams (standard tanks and ambient air) at each of the station. The standards are analyzed by the system every 6 to 12 hours. Similarly, the flask measurements in Beijing lab are also linked to the WMO/GAW standards to guarantee the comparability in the community.

Data processing

For the continuous in-situ CH_4 observation systems, raw data from the instrument are collected by the data acquisition system. Then they are identified by different gas streams and separated into 5 minutes clusters. The data processing routine dumps the first 3 minutes data and only uses the last 2 minutes average to calculate the concentration through the latest linearity regression factors. Ambient CH_4 measurements are only included in the final data set if the re-evaluation of target gas relative to the WH and WL is within 4 ppb of its assigned value. The data out of this criterion are flagged by principal investigator and are deemed invalid. The data are also manually inspected and examined using quality control routines before being accepted as valid measurements. After these steps, all of the 5-minute data were combined into hourly averages and used for further analysis.

Data filtering

All CMA stations except WLG locate in regions with intense human activities, CH_4 observations at these sites are unavoidably influenced by local contaminations. To understand the CH_4 level on a regional scale, we filter the data into “regional” and “local” events. The “regional” events represent CH_4 mole fractions on a large regional scale (generally > 10 km^2), while the “local” events represent values heavily influenced by sources near the station (≤ 10 km^2). Only CH_4 measurements flagged as “regional” are used in our inversion. Figure S11 compares “regional” and “local” hourly/weekly mean observations.

The data filtering follows a protocol developed in our previous study [1] and is described briefly as follows.

For continuous (hourly) measurements, we only use data during daylight hours, e.g., 9:00 to 17:00 (local time) at LAN and from 9:00 to 16:00 (local time) at LFS and SDZ. Then, CH_4 observations are determined to be locally representative and are discarded when surface winds are from potential local sources such as nearby villages, factories, paddy rice fields. To minimize the influence of very local sources / sinks, we further discard the data when the local surface wind speed is lower than 1.5 m s^{-1}. Additionally, at WLG station, we also flag the CH_4 records when the wind speed is higher than ≥ 10 m s^{-1}, because the higher wind speed may also transport emissions from broader regions (e.g., Xining and Lanzhou) at WLG. Finally, the remainder of data is considered regionally representative and are used in our study.

For the discrete flask samples at JSA and AKD, anomalous data caused by improper sample collection operations [4] or error in analysis (flagged by QC), are initially rejected. Then, paired samples with differences larger than 5 ppb are regarded as contamination of one or both flasks and rejected. This value (5 ppb) corresponds to three times the average pair agreement. After these processing, the averages of each pair of flask samples are used in this study.

Text S2 Impact of surface observations on the inversion

To evaluate the impact of surface observations on our inferences, we perform inversions with only GOSAT satellite observations and compare to our main inversions. Both inversion systems can reduce errors against observations, relative to the prior simulation (Table S6) and produce results that are generally consistent (Figure S3). Compared to the satellite-only inversion, our main inversion (satellite+surface) estimates a smaller national total emission (satellite+surface: 54 Tg a^{-1}; satellite-only: 59 Tg a^{-1}) but a larger 2010–2017 emission trend (satellite+surface:0.73 Tg a^{-2}; satellite-only: 0.16 Tg a^{-2}). Regionally, differences in mean emissions occurs mainly over Northeast, while the largest differences in trends are found in Northeast and East China (Figure S3 and Figure S4).

These differences discussed above reflect additional information gained from surface observations. Figure S3 and Figure S4 show that the addition of surface observations improves observational constraints for both mean emissions and emission trends. The number of independent pieces of information (DOFS) from the inversion increases from 114 (44) to 146 (65) for mean emissions and increase from 44 (18) to 63 (30) for inter-annual trends over the East Asia domain (over China). The largest improvement occurs in Northeast and East China, where satellite measurements alone have only weak constraints because of sparse sampling owing to frequent unfavorable conditions (e.g., cloud, snow surfaces, and low solar zenith angle). DOFS are more than doubled for resolving inter-annual trends over Northeast (from 1 to 3) and East (from 2 to 4) China (Figure S3). Analysis of footprints for individual sites shows that the additional constraints are mainly provided by LFS for Northeast and LAN and JSA for East (Figure S6 and Figure S7). These results demonstrate the value of including surface observation in our inversion.

Text S3 Effect of straw incorporation on rice methane emissions

Incorporation of straw into crop fields is considered a favorable management practice of crop residue utilization. Straw incorporation improves soil fertility and thus increase crop yields. However, it also increases methane emissions significantly from rice paddies [5-7], by stimulating methanogen activities with enhanced organic input [8]. A meta-analysis study estimates that straw incorporation increases methane emissions from rice paddies by 110% [5], although it is found that this effect gradually reduces over a long period of time (> 5 years) [7].

It is estimated that China generates ~800 million tonne of crop residues every year [9]. Traditionally, a large fraction of them is burned in field, causing serious problems of air pollution and nutrition loss. Crop residue utilization has been advocated by the policy of the Chinese government. Incorporating straw as organic fertilizer is a major approach to utilize crop residues, among other approaches such as conversion to heating fuels and livestock forage. In the last decade, the government has implemented increasingly stricter straw burning ban, as part of the clean air campaign. Consistent with this policy, during the last decade, the crop residue utilization rate increases rapidly in China, with straw incorporation into fields being a major contributor.

This drastic change in agricultural practices is not accounted for in bottom-up modeling of rice emissions, resulting in almost a zero trend from the rice cultivation sector in all of our prior inventories. Here we estimate the effect of increasing straw incorporation following the methodology outlined by Yan, et al. [10]. We collect annual province-level rice cultivation areas (including early rice, middle rice, and late rice) for 2010–2017 from the agricultural yearbook (Figure S9). We use methane emission factors as a function of region, rice season, water regime, and organic input tabulated by Yan, et al. [10], which assumes that application of organic inputs (e.g., straw incorporation) doubles the emission factor. We assume 66.7% of rice paddies under intermittent irrigation while the rest 33.3% under continuous flooding [10]. To quantify the effect of increasing straw incorporation, we parameterize annual-varying and region-dependent straw incorporation fractions, using values taken from two studies in China [9,11] (Figure S10). In comparison, Yan, et al. [10] assumed a constant 50% of rice paddies receive organic inputs (30% from crop straws and 20% from animal and human wastes).

Our bottom-up calculation yields an average national rice methane emission of 7.7 Tg a^{-1} and a linear emission trend of 0.08 Tg a^{-2} for 2010–2017, with the largest contributors to the trend being Central and East China. These bottom-up trends are comparable to those inferred from our inversions. Comparison to a sensitivity calculation assuming a constant regional organic input fraction indicates that the positive trends in Central and East China are driven by the increase in straw incorporation, while that in Northeast is also contributed by the increase in the rice cultivation area. Our results suggest that the positive methane emission trends inferred from the inversion can largely be explained by increased amount of straw incorporation in the last decade, in response to the strict implementation of environmental regulations.

Text S4 Freshwater aquaculture methane emissions in China

China is the largest producer of freshwater aquaculture in the world, accounting for > 60% of the global production by weight [12]. Like rice paddies, the anoxic and carbon-rich condition of freshwater aquaculture is favorable for methane emissions [13], making freshwater aquaculture a uniquely important methane emitting sector in China. This sector is currently underrepresented in most methane emission inventories including those used in our inversion. Moreover, statistics show that China’s freshwater aquaculture industry continues to grow in terms of its volume and area in recent years, accompanied by conversion of rice paddies to aquaculture ponds and expansion of rice-fish systems. Given the great spatial overlap between rice paddies and freshwater aquaculture, it is possible that freshwater aquaculture contributes to the positive methane emission trends over the rice producing regions inferred by our inversion.

Here, we make a rough estimation of methane emissions from the freshwater aquaculture sector. Following Yuan, et al. [13], we classify freshwater aquaculture as rice-fish systems, extensive plus semi-intensive systems, and intensive systems. Yuan, et al. [13] reported emissions factors of 4.5 mg CH_4 m^{-2} h^{-1} for rice-fish systems, 4.8 mg CH_4 m^{-2} h^{-1} for extensive plus semi-intensive systems, and zero methane emissions for intensive systems. We then applied these emission factors to annual province-level freshwater aquaculture areas for the two systems collected from the China Fishery Yearbook (Figure S9).

Our bottom-up calculation yields a national emission of 3.0 Tg a^{-1} from freshwater aquaculture, with a positive trend of 0.02 Tg a^{-2} mainly driven by Central China. Our results suggest that freshwater aquaculture is a nonnegligible sector of methane emissions in China, but its trend is unlikely to explain the observed increase in methane emissions from East and Central China.

Text S5 Construction of S_O

The observation error covariance matrix ($\mathbf{S}_{\mathrm{O}}$) specifies the error structure of the observation vector ($\boldsymbol{y}$) including contributions from instrument errors and model errors. Observation errors can be determined empirically from the statistics of residual errors [14], which is defined as the random component of model-observation differences and are computed as $\varepsilon_O = \boldsymbol{y} - \boldsymbol{y}_a - \overline{\boldsymbol{y} - \boldsymbol{y}_a}$ (overbar denotes annual averages in 0.5°×0.625° grid cells). Table S4 tabulates thus derived variance (σ^2), spatial error correlation scales (ρ_s), and temporal error correlation scales (ρ_t), computed separately for GOSAT observations and individual surface sites. We find an average variance of 13.4^2 ppbv^2, a spatial correlation scale of 250 km, and a temporal correlation scale of 14 days for satellite observations. For different surface sites, we find error variances ranging from 18^2 ppbv^2 in WLG to 82^2 ppbv^2 in LAN, generally reflecting increasing model errors from clean-background sites to near-source sites. We find no (spatial) correlation between different sites but a temporal correlation scale of roughly 2 days for surface observations.

With this information, we can specify a full error covariance matrix (denoted as $\mathbf{S}_{\mathrm{O}}'$). $\mathbf{S}_{\mathrm{O}}'$ can be decomposed as $\mathbf{S}_{\mathrm{O}}' = \mathbf{\Lambda}^{\frac{1}{2}}\mathbf{\Sigma}\mathbf{\Lambda}^{\frac{1}{2}}$, where $\mathbf{\Lambda}$ is the variance matrix (diagonal) and $\mathbf{\Sigma}$ is the error correlation matrix. Diagonal entries of $\mathbf{\Lambda}$ are populated with variance (σ^2) corresponding to each observation, while entries of $\mathbf{\Sigma}$ (r_{ij}) are populated with

$$r_{ij} = \exp\left(-\frac{d}{\rho_s}\right)\exp\left(-\frac{t}{\rho_t}\right), \tag{S1}$$

where *d* and *t* are difference in distance and time between two observations. We specify $r_{ij} = 0$ between a pair of satellite and surface observations and between surface observations from different sites.

However, computing the inverse of this full error covariance matrix (${\mathbf{S}_{\mathrm{O}}'}^{-1}$), required in Eq. (2), is computationally intractable for a large number of observations (~ 400,000 in our study), because its algorithmic complexity is $O(n^3)$. For this reason, inversion studies often approximate the full matrix $\mathbf{S}_{\mathrm{O}}'$ as a diagonal matrix $\mathbf{S}_{\mathrm{O}} = \gamma^{-1}\mathbf{\Lambda}$, essentially neglecting the error correlation structure ($\mathbf{\Sigma}$) [15-17]. A regularization parameter γ (often < 1) is introduced to inflate $\mathbf{\Lambda}$, to prevent overfitting that results from neglecting $\mathbf{\Sigma}$. Determination of γ can be based on the corner of a L-curve plot [18]. However, the L-curve plot may not show a clearly defined corner, or sometimes, multiple corners, making it difficult to objectively choose γ [17].

Below, we develop a method to analytically determine the value of γ. We define the difference between $\gamma\mathbf{\Sigma}$ and the identity matrix $\mathbf{I}$ in the sense of the Frobenius norm:

$$L = ||\gamma\mathbf{\Sigma} - \mathbf{I}||_F^2, \tag{S2}$$

where the Frobenius norm of a matrix **A** is $||\mathbf{A}||_F^2 = tr(\mathbf{A}\mathbf{A}^T) = \sum_i \sum_j a_{i,j}^2$ (*tr* is the trace of a matrix). Eq.(S2) can be expanded:

$$L = tr(\mathbf{\Sigma}\mathbf{\Sigma}^T)\gamma^2 - 2tr(\mathbf{\Sigma})\gamma + tr(\mathbf{I}) = ||\mathbf{\Sigma}||_F^2\gamma^2 - 2n\gamma + n\,, \tag{S3}$$

where *n* is the dimension of $\mathbf{S}_{\mathrm{O}}$ (i.e., number of observations). Minimization of *L* is achieved when $\frac{dL}{d\gamma} = 0$, which yields the optimal value of γ:

$$\gamma = \frac{n}{\|\boldsymbol{\Sigma}\|_F^2} = \frac{n}{\sum_i \sum_j r_{i,j}^2} \tag{S4}$$

The complexity of computing Eq. (S4) is also $O(n^3)$ for a full error correlation matrix $\boldsymbol{\Sigma}$. However, in practice, computation of $\sum_i \sum_j r_{i,j}^2$ can readily be parallelized and $\boldsymbol{\Sigma}$ is often sparse (correlations between spatially and/or temporally distant observations are essentially 0). Moreover, if observations are generally evenly distributed spatially and temporally (as in the case of GOSAT data), just computing the average of a random subset of $r_{i,j}^2$ is sufficient for a good estimation of $\sum_i \sum_j r_{i,j}^2$.

We compute the regularization parameters using Eq. (S4) separately for satellite and surface observations, because of their distinct error correlations (Table S4). This yields $\gamma = 0.18$ for GOSAT observations, $\gamma = 1$ for weekly surface observations, and $\gamma = 0.7$ for daily surface observations. These choices of γ is comparable to a previous high-resolution regional inversion for North America [19,20].

Text S6 Inversion uncertainties and observational constraints

We evaluate the uncertainty of national and regional inversion results by analyzing (1) the posterior error covariance matrix ($\hat{\mathbf{S}}$) and (2) the sensitivity of results to perturbed prior emission inventories (Table S7). We also analyze the averaging kernel matrix of the inversion to show the spatial distribution of observational constraints.

The posterior covariance matrix ($\hat{\mathbf{S}}$) describes the error covariance structure of the posterior estimates for the state vector of the inversion. The closed-form solution of $\hat{\mathbf{S}}$ is given as

$$\hat{\mathbf{S}} = (\mathbf{K}^{\mathrm{T}}\mathbf{S}_{\mathrm{O}}^{-1}\mathbf{K} + \mathbf{S}_{\mathrm{a}}^{-1})^{-1}, \quad (S5)$$

where $\mathbf{S}_a$ is prior errors and $\mathbf{S}_O$ is observation errors. Overall, $\hat{\mathbf{S}}$ tends to provide an optimistic estimate of uncertainties. This is because error structures of $\mathbf{S}_a$ and $\mathbf{S}_O$ (Eq. (S5)) are often inadequately represented in inversions for computational reasons and for lack of better information [15]. In addition, **S** describes only random errors (precision) of the inversion by definition; systematic errors (e.g., in the transport model) or accuracy are thus not accounted for. Nevertheless, the lower bound estimates provided by analyzing $\hat{\mathbf{S}}$ are still useful. We compute the error covariances ($\hat{\mathbf{S}}_{\mathrm{agg}}$) for spatially aggregated estimates of emissions and their trends ($\hat{\boldsymbol{x}}_{\mathrm{agg}}$):

$$\hat{\mathbf{S}}_{\mathrm{agg}} = \mathbf{W}\hat{\mathbf{S}}\mathbf{W}^{\mathrm{T}}, \quad (S6)$$

where **W** is the transformation matrix that $\hat{\boldsymbol{x}}_{\mathrm{agg}} = \mathbf{W}\hat{\boldsymbol{x}}$. Table S7 tabulates standard deviations of regional estimates based on Eq. (S6).

In addition, we evaluate uncertainties arising from prior emission distributions. Studies have shown that falsely allocated prior emissions may cause biases in flux estimates and, more importantly, sector attribution [21]. We therefore perturb our prior anthropogenic emissions with an ensemble of emission inventories. We then report the means and the ranges of estimations from this inversion ensemble (Table S2 and Table S7). Figure 1C and 1D show that ranges of posterior estimates are greatly reduced from those of prior estimates for regional and national total emissions and their trends, suggesting strong regional constraints on total emissions from the observations. In comparison, ensemble ranges of sectoral emissions are not always reduced from prior to posterior estimates on the regional and national levels (Figure 2), which reflects additional uncertainties from prior emission distributions in sector attribution.

The degree to which inversion results are constrained by observations can be quantitively described by the averaging kernel matrix (**A**), which is given by the following equation:

$$\mathbf{A} = \mathbf{I} - \hat{\mathbf{S}}\mathbf{S}_{\mathrm{a}}^{-1}. \quad (S7)$$

The diagonal terms of **A**, referred to as averaging kernel sensitivities, measures the ability of the observations to constrain individual state vector elements, and the sum of averaging kernel sensitivities, referred to as degrees of freedom for signal (DOFS), quantifies the number of independent pieces of information constrained by the inversion. Our inversion (using both satellite and surface measurements) achieves 146 and 63 DOFS for mean methane emissions and inter-annual anomalies. Figure S5 plots the spatial distribution of averaging kernel sensitivities corresponding to 2010–2017 mean methane emissions and their inter-annual anomalies, respectively. Figure S3 shows aggregated DOFS for regions in China and Table S2 tabulates that

for each province. Overall, the observations provide reasonably good constraints over major regions in eastern China, except for southern China (i.e., Guangdong, Guangxi, and Hainan provinces), which is not well covered by either satellite or surface observations.

Table S1 Inversion-based estimates of China's anthropogenic emissions and trends.

	Average emissions ($Tg\ a^{-1}$)	Trends ($Tg\ a^{-2}$)	Time range	Observation	Transport model [a]
Bergamaschi 2013 [22]	40–50	1.1±0.3	2000–2010	SCIAMACHY + surface	TM5 (4°×6°)
Thompson 2015 [23]	54 ± 4 [b]	1.21±0.43 [b]	2000–2011	Surface	FLEXPART
Miller 2019 [24]	59	1.1±0.4 [b]	2010–2015	GOSAT	GEOS-Chem (2°×2.5°)
Wang 2019 [25]	44–48	—	2010–2012	GOSAT+ surface	NIES-TM (2.5°×2.5°) + FLEXPART
Janardanan 2020 [26]	46±9	—	2011–2017	GOSAT+ surface +ship +aircraft	NIES-TM (2.5°×2.5°) + FLEXPART
Saunois 2020 [27]	40±3 (2000-2009)<br>50±8 (2008-2017)	1.3 [c]	2000–2017	Surface or GOSAT	Ensemble of 19 inversions<br>Varied transport models
Lu 2021 [17]	43	0.1	2010–2017	GOSAT + surface +ship +aircraft	GEOS-Chem (4°×5°)
Qu 2021 [28]	56	—	2019	GOSAT	GEOS-Chem (2°×2.5°)
Sheng 2021 [21]	55 ± 2	0.36±0.04 [b]	2010–2017	GOSAT + surface	UK Met Office NAME
Wang 2021 [29]	54±9 (2009-2013) [b]<br>62±9 (2014-2018) [b]	1.7 [b,c]	2009–2018	GOSAT+ surface	NIES-TM (2.5°×2.5°) + FLEXPART
Yin 2021 [30]	—	1.0±0.2	2010–2017	GOSAT + surface +ship +aircraft	LMDz-INCA (1.875°×3.75°)

Zhang 2021 [31]	47±1	0.39±0.27	2010–2018	GOSAT	GEOS-Chem (4°×5°)
Stavert 2022 [32]	45(41-55)	—	2008–2017	GOSAT + surface	Ensemble of 22 inversions<br>Varied transport models

[a] Grid resolutions for Lagrangian transport models are listed.

[b] Total emissions instead of anthropogenic emissions are reported.

[c] Trend estimated from reported average emissions of two time periods.

Table S2 Posterior estimates of methane emissions and trends by province [a].

Province	Average Emissions (Tg a^{-1})	Average emissions DOFS	Emission Trends ($\times 10^{-2}$ Tg a^{-2})	Emission Trends DOFS
Shanxi	5.2 [4.7 – 5.7]	3.1/5.2	4.1 [3.4 – 5.1]	2.6/5.2
Sichuan	3.1 [3.1 – 3.2]	3.6/15.4	-1.5 [-4.8 – 0.0]	1.4/15.4
Guizhou	3.0 [2.5 – 3.7]	1.0/5.1	-3.3 [-6.7 – -1.6]	0.5/5.1
Yunnan	2.7 [2.5 – 3.0]	2.2/9.1	-3.1 [-4.0 – -1.5]	0.9/9.1
Hunan	2.4 [2.2 – 2.6]	1.8/8.1	3.1 [-1.2 – 6.5]	0.6/8.1
Anhui	2.3 [2.3 – 2.4]	2.5/7.4	3.1 [2.2 – 3.8]	1.1/7.4
Guangxi	2.2 [1.8 – 2.4]	1.2/9.0	0.6 [0.1 – 1.2]	0.3/9.0
Guangdong	2.1 [2.0 – 2.3]	1.2/11.0	-0.6 [-1.5 – 0.1]	0.3/11.0
Hubei	2.1 [1.7 – 2.4]	2.2/8.2	1.9 [1.4 – 2.4]	0.8/8.2
Shandong	2.0 [1.8 – 2.3]	2.6/9.5	1.9 [1.5 – 2.1]	1.1/9.5
Neimenggu	2.0 [1.7 – 2.7]	4.5/17.8	5.8 [2.2 – 12.8]	2.6/17.8
Henan	2.0 [1.7 – 2.2]	2.7/9.5	0.4 [-6.5 – 4.9]	1.1/9.5
Jiangsu	1.9 [1.6 – 2.2]	2.7/8.1	6.5 [4.5 – 7.8]	1.3/8.1
Jiangxi	1.9 [1.8 – 2.0]	1.5/5.9	5.3 [4.3 – 6.3]	0.6/5.9
Shaanxi	1.7 [1.2 – 2.6]	2.0/5.6	7.6 [4.3 – 16.8]	1.0/5.6
Heilongjiang	1.6 [1.5 – 1.7]	2.8/11.6	13.3 [9.0 – 16.8]	1.0/11.6
Liaoning	1.5 [1.4 – 1.6]	2.0/6.1	5.0 [0.7 – 7.3]	0.8/6.1
Zhejiang	1.5 [1.2 – 1.6]	2.3/5.1	4.5 [2.5 – 5.7]	1.4/5.1
Xinjiang	1.4 [1.2 – 1.5]	5.2/27.4	0.4 [-1.3 – 3.8]	2.1/27.4
Chongqing	1.3 [1.2 – 1.4]	1.1/3.3	-0.5 [-3.9 – 1.0]	0.4/3.3
Hebei	1.3 [1.1 – 1.4]	3.5/11.1	2.7 [1.6 – 3.9]	1.5/11.1

Fujian	1.0 [0.8 – 1.1]	0.5/4.4	0.5 [-0.9 – 1.1]	0.1/4.4
Jilin	0.9 [0.8 – 1.1]	2.0/5.4	3.6 [2.4 – 4.2]	1.0/5.4
Xizang	0.5 [0.5 – 0.6]	1.2/20.4	-0.1 [-0.7 – 0.9]	0.3/20.4
Shanghai	0.4 [0.3 – 0.4]	0.7/1.3	0.3 [-0.1 – 0.8]	0.4/1.3
Ningxia	0.4 [0.3 – 0.4]	0.5/1.1	1.2 [0.8 – 2.3]	0.2/1.1
Gansu	0.3 [0.3 – 0.4]	2.2/9.2	3.0 [2.2 – 3.6]	0.9/9.2
Hainan	0.3 [0.3 – 0.4]	0.1/1.2	0.0 [-0.4 – 0.1]	0.0/1.2
Qinghai	0.3 [0.2 – 0.3]	3.7/13.1	0.9 [0.6 – 1.1]	2.2/13.1
Beijing	0.2 [0.2 – 0.3]	1.4/3.0	1.4 [0.6 – 2.2]	1.1/3.0
Tianjin	0.2 [0.2 – 0.2]	0.7/1.6	0.7 [0.6 – 0.9]	0.3/1.6
Taiwan	0.2 [0.1 – 0.2]	0.8/3.7	-0.5 [-0.5 – -0.3]	0.2/3.7

[a] Posterior estimates of 2010-2017 average emissions and emission trends are shown as ensemble means and ranges (mean [min – max]). Ensemble average degrees of freedom for signals (DOFS) of average emissions and trends are also shown, together with the number of spatial groups that are optimized in each province (DOFS / # of spatial groups). The table does not include Hong Kong and Macau as they are not resolved by the 0.5°×0.625° grid.

Table S3 Surface site information for the CMA network and the WDCGG network.

Network	Site	Longitude	Latitude	Altitude	Measurement
CMA [a]	WLG	100.09	36.12	3816	Hourly in situ (PICARRO)
	LAN	119.72	33.30	138.6	Hourly in situ (PICARRO)
	LFS	127.60	44.73	330.5	Hourly in situ (PICARRO)
	XGL	99.40	28.00	3580	Hourly in situ (PICARRO)
	SDZ	117.12	40.65	293.3	Hourly in situ (PICARRO)
	AKD	87.93	47.10	562	Weekly flask
	JSA	114.22	29.63	750	Weekly flask
WDCGG [b]	AMY	126.33	36.539	128.0	Hourly In situ
	RYO [c]	141.82	39.032	280.0	Hourly In situ
	PDI [d]	103.52	21.573	1478.0	Hourly In situ
	TAP [e]	126.13	36.738	21.0	Weekly flask
	LLN [e]	120.87	23.470	2867.0	Weekly flask
	UUM [e]	111.10	44.452	1012.0	Weekly flask

[a] China Meteorology Administration data used in this study are available at a public data repository (https://doi.org/10.57760/sciencedb.02269). All sites are available during 2010–2017 except for XGL which is unavailable in 2013.

[b] Data from the World Data Center for Greenhouse Gas (WDCGG) are accessed through https://gaw.kishou.go.jp/. All sites are available during 2010–2017 except for PDI which starts in 2014.

[c] DOI for RYO hourly in situ data: 10.50849/WDCGG_0001-2012-1002-01-01-9999

[d] DOI for PDI hourly in situ data: 10.50849/WDCGG_0051-2035-1002-01-01-9999

[e] DOI for weekly flask data at TAP, LLN, and UUM: 10.15138/VNCZ-M766

Table S4 Error variances and correlation scales derived from the statistics of residual errors.

Observations		Standard deviation (ppbv) [a]	Temporal correlation scale (day) [b]	Spatial correlation scale (km) [b]
Satellite – GOSAT		13.4 [c]	14	250
CMA surface observations	WLG	18	2	N/A
	LFS	78		
	XGL	26		
	SDZ	80		
	AKD	31		
	JSA	70		
WDCGG surface observations	AMY	55		
	RYO	18		
	PDI	38		
	TAP	59		
	LLN	24		
	UUM	27		

[a] Error standard deviations are directly computed from residual errors for each surface sites and for each model grid cell if the number of GOSAT observations in that grid cell is greater than 10 per year. For grid cells observed <10 times a year by satellite, the domain average error standard deviation for satellite observations (13.4 ppbv) is used.

[b] Error correlation scales are fitted as the exponents of an exponential function as a function of distance or time using residual errors. A single set of values are derived for either satellite or surface observations. This analysis finds no significant spatial correlation between surface sites, most of which are well separated spatially.

[c] Error standard deviations for satellite observations vary spatially. The 5th to 95th percentile range is 10 to 20 ppbv, with an average of 13.4 ppbv.

Table S5 Prior anthropogenic emission inventories used in the inversion ensemble.

**Name**	**Data Source**	**Emissions** ($Tg\ a^{-1}$)	**Trend** ($Tg\ a^{-2}$)
E1	EDGAR v4.3.2 for 2012, except for coal (Sheng et al., 2019)	59	0
E2	PKU_CH_4 v2 2010–2017	49	-0.3
E3	EDGAR v5.0 2010–2015	60	+0.4
E4	CEDS v2021-04-21	51	+0.2

Table S6 Fitness of prior and posterior simulations to satellite and surface observations.

		**Mean Bias (ppbv)**	**Root mean square error (ppbv)**	**Correlation**
GOSAT observations	Prior	-2.4	16	0.90
	Posterior (satellite+surface)	-1.6	14	0.92
	Posterior (satellite only)	-1.0	14	0.92
Surface Observations	Prior	16	51	0.73
	Posterior (satellite+surface)	0.2	35	0.82
	Posterior (satellite only)	14	46	0.75

Table S7 Prior and posterior uncertainties for national and regional estimates [a].

	Mean emissions ($Tg\ a^{-1}$)		Emission trends ($Tg\ a^{-2}$)	
	Prior	Posterior	Prior	Posterior
Error SD computed from error covariance matrix				
Northeast	0.6	0.4	0.10	0.06
North	2.0	1.0	0.30	0.07
East	0.9	0.5	0.15	0.07
Central	0.9	0.5	0.14	0.09
Southwest	1.3	0.7	0.21	0.09
China	3.0	1.6	0.47	0.15
SD and half range derived from the inversion ensemble[b]				
Northeast	0.7 [0.8]	0.2 [0.2]	0.07 [0.07]	0.06 [0.07]
North	2.9 [3.3]	0.2 [0.2]	0.08 [0.09]	0.01 [0.01]
East	2.0 [2.1]	0.1 [0.1]	0.03 [0.04]	0.02 [0.02]
Central	1.5 [1.5]	0.2 [0.2]	0.05 [0.06]	0.04 [0.04]
Southwest	2.4 [2.7]	0.3 [0.3]	0.13 [0.14]	0.05 [0.06]
China	5.7 [5.7]	0.3 [0.4]	0.30 [0.35]	0.12 [0.14]

[a] Uncertainties of regional estimates are evaluated based on the error covariance matrix and the inversion ensemble, respectively. See Text S6 for more descriptions on two methods of uncertainty evaluation.

[b] Standard deviations and half ranges (in bracket) are computed based on inversion results of 4 ensemble members.

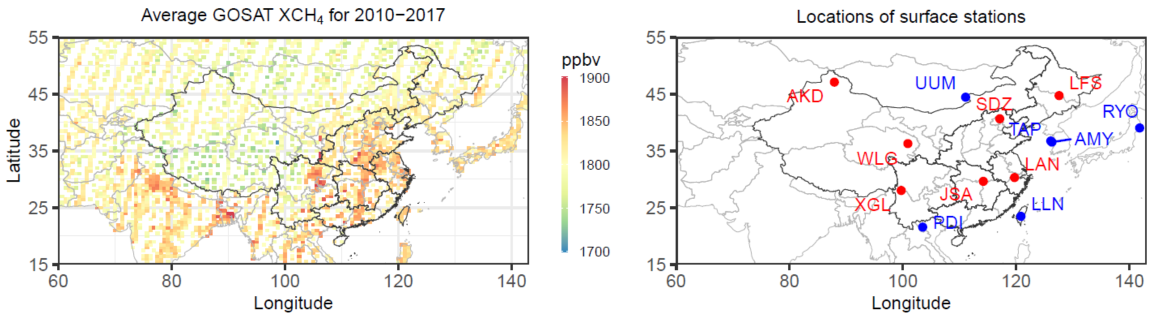

Figure S1. Satellite and surface methane observations. (Left) Distribution of average GOSAT column methane mixing ratios for 2010–2017; (Right) Locations of surface sites. Sites in the CMA network is shown in red, and sites in the WDCGG network is in blue.

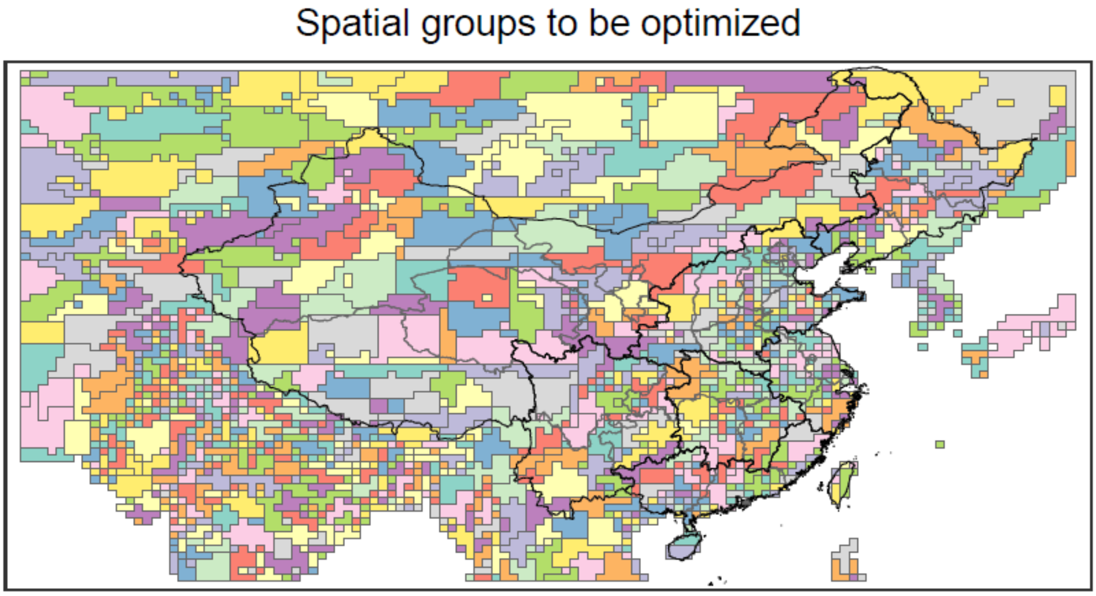

Figure S2. 600 spatial groups on which methane emissions are optimized, generated based on a Gaussian Mixture Model. Filled colors are to help differentiate these groups.

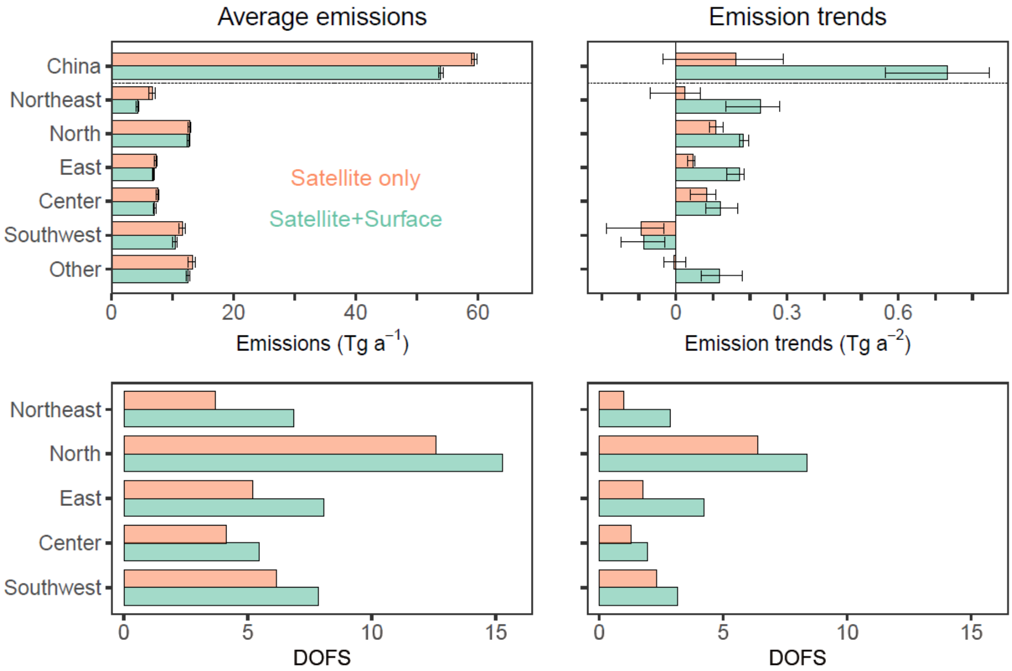

Figure S3. Comparison of regional and national posterior estimations between inversions using only satellite observations and those using both satellite and surface observations. (Top) Nationally or regionally aggregated inversion results; (Bottom) Observational constraints shown as DOFS. (Left) 2010–2017 average emissions; (Right) 2010–2017 emission trends. Error bars represent ranges from the inversion ensemble. Variations in DOFS among ensemble members are negligible and therefore corresponding error bars are not plotted.

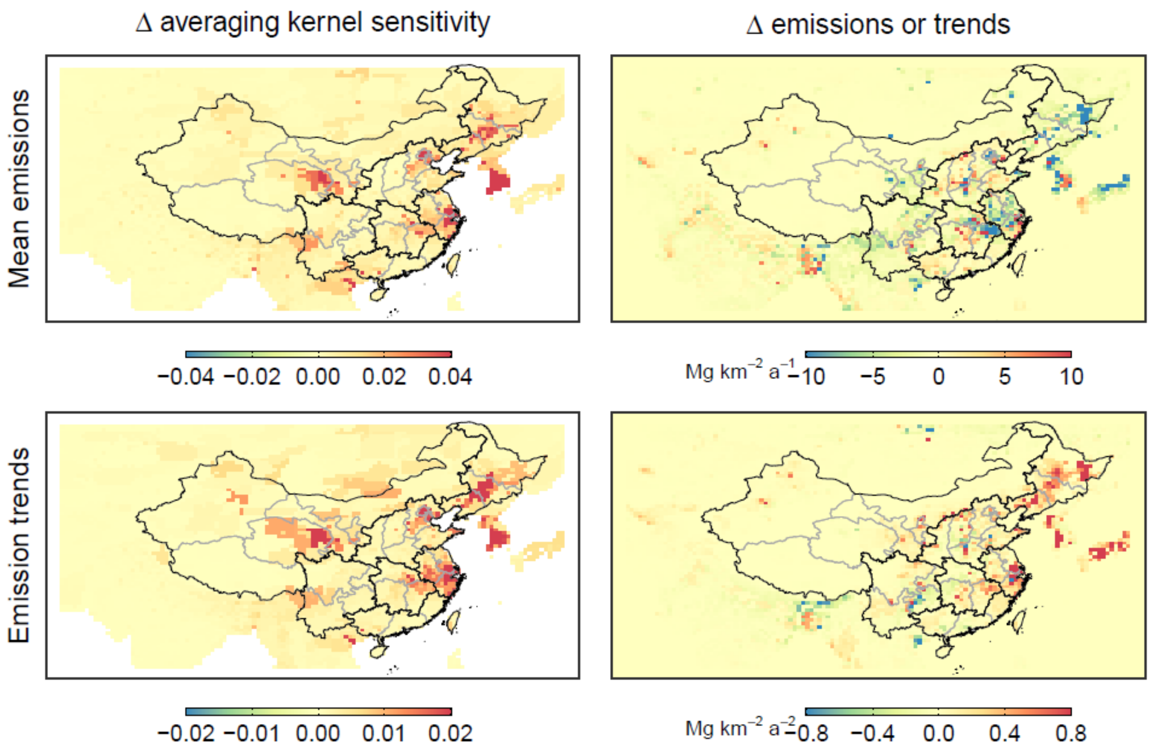

Figure S4. Impact of including surface observations on the inference of mean emissions (top) and emission trends (bottom). Left column shows the difference in averaging kernel sensitivity and right column inferred mean emissions or emission trends. Differences are computed as the satellite+surface inversion minus the satellite-only inversion.

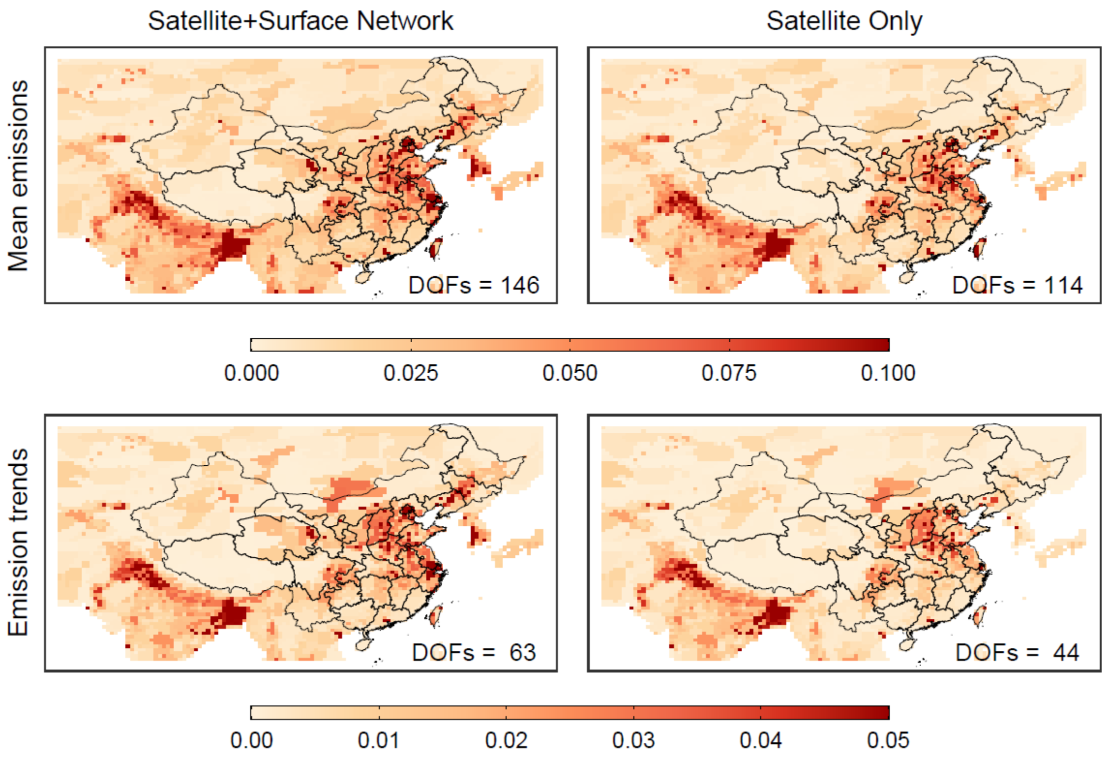

Figure S5. Averaging kernel sensitivities of the inversions for mean emissions (Top) and emission trends (Bottom). Original averaging kernel sensitivities for 600 emission clusters are projected to 0.5°×0.625° simulation grid cells with conservation of DOFS. Larger values represent better constraints by observations. Left panels are for inversions using both satellite and surface observations, and right panels for inversions using only satellite observations. DOFS for each inversion are inset.

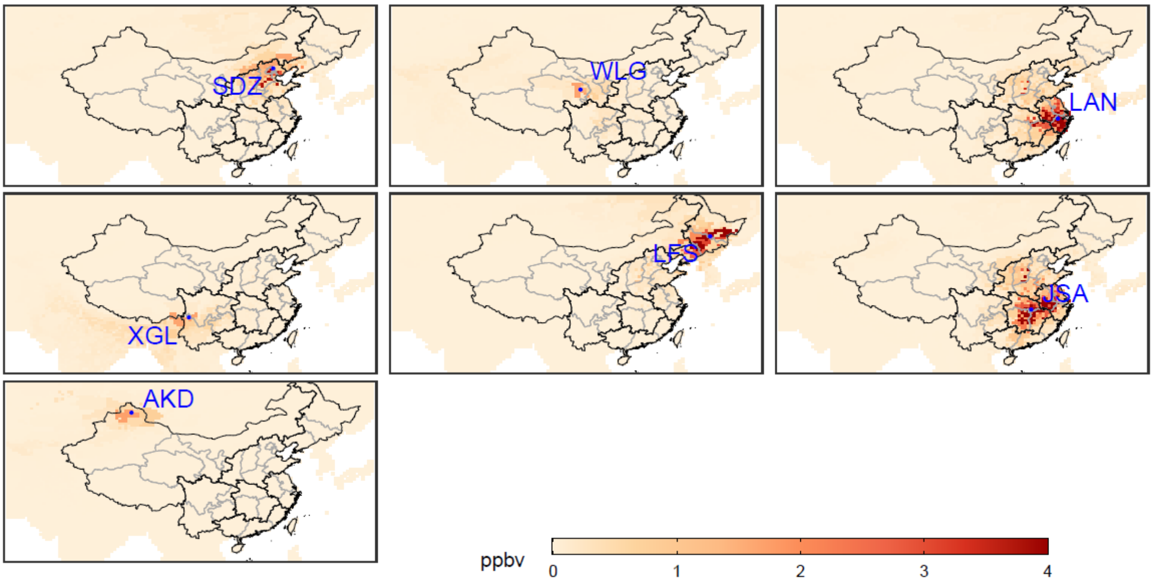

Figure S6. Average sensitivities (footprint) of surface observations at CMA sites to 100% perturbation of prior emissions from different locations.

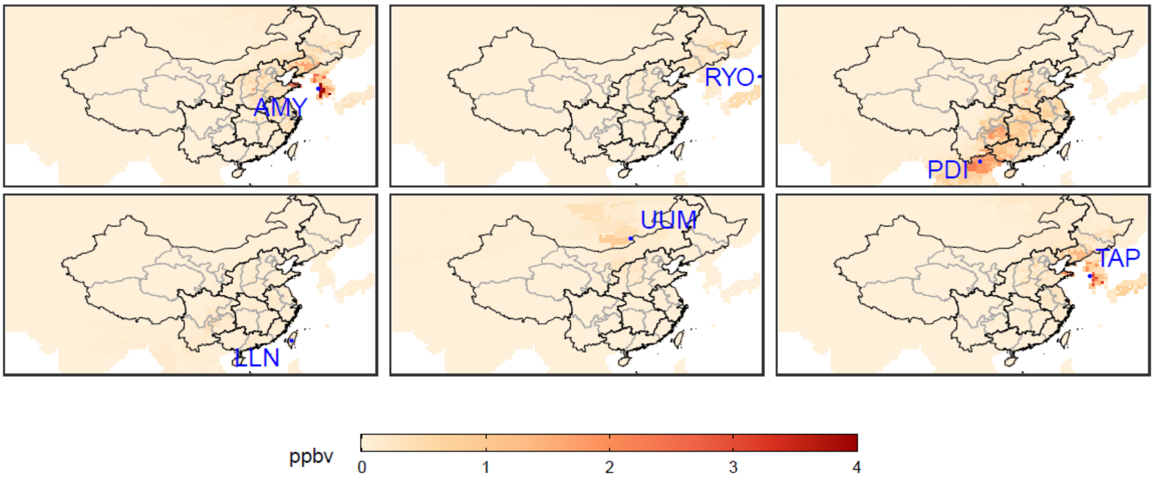

Figure S7. Average sensitivities (footprint) of surface observations at WDCGG sites to 100% perturbation of prior emissions from different locations.

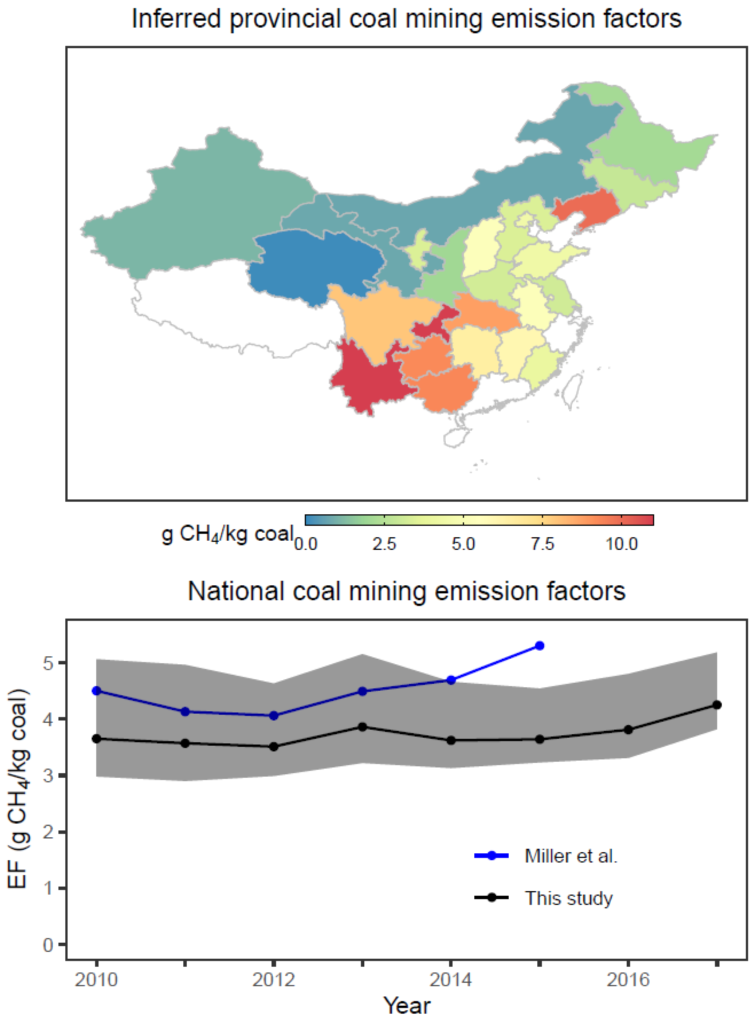

Figure S8. Methane emission factors from coal mining. (Top) Provincial coal mining emission factors inferred from the inversion results. (Bottom) Nationally averaged coal mining emission factors from 2010–2017.

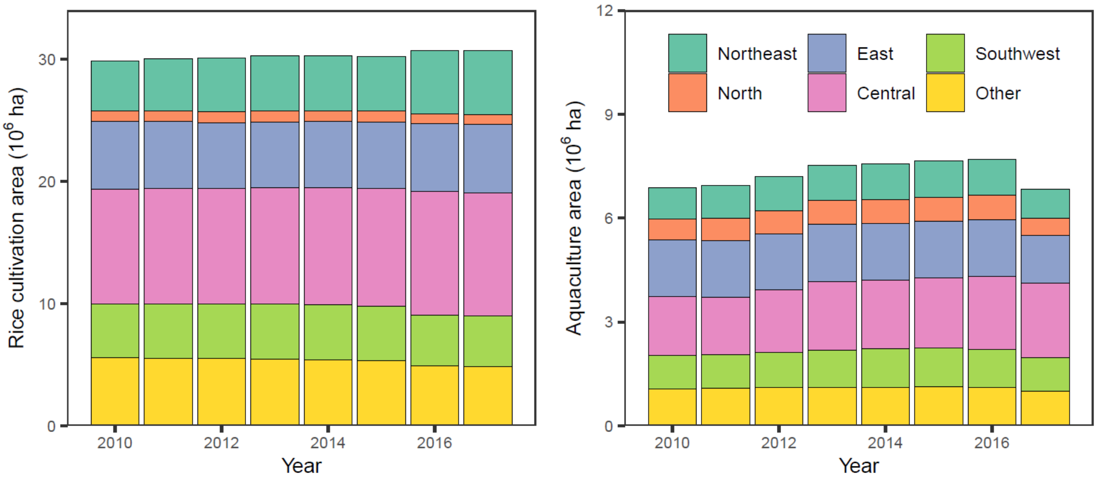

Figure S9. Rice cultivation areas (Left) and freshwater aquaculture areas (Right) in China from 2010–2017. Color shadings represent regions. Data for rice cultivation areas are taken from China Agriculture Statistical Yearbook[33] and data for freshwater aquaculture areas from China Fishery Statistical Yearbook[34].

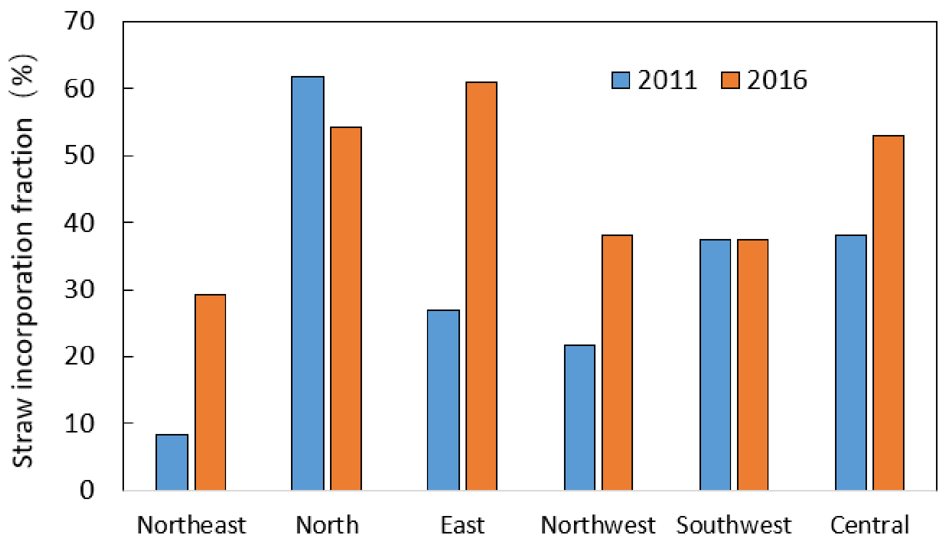

Figure S10. Fractions of straw retained in crop fields in different regions of China in 2011 and 2016. Data are derived from [9,11]

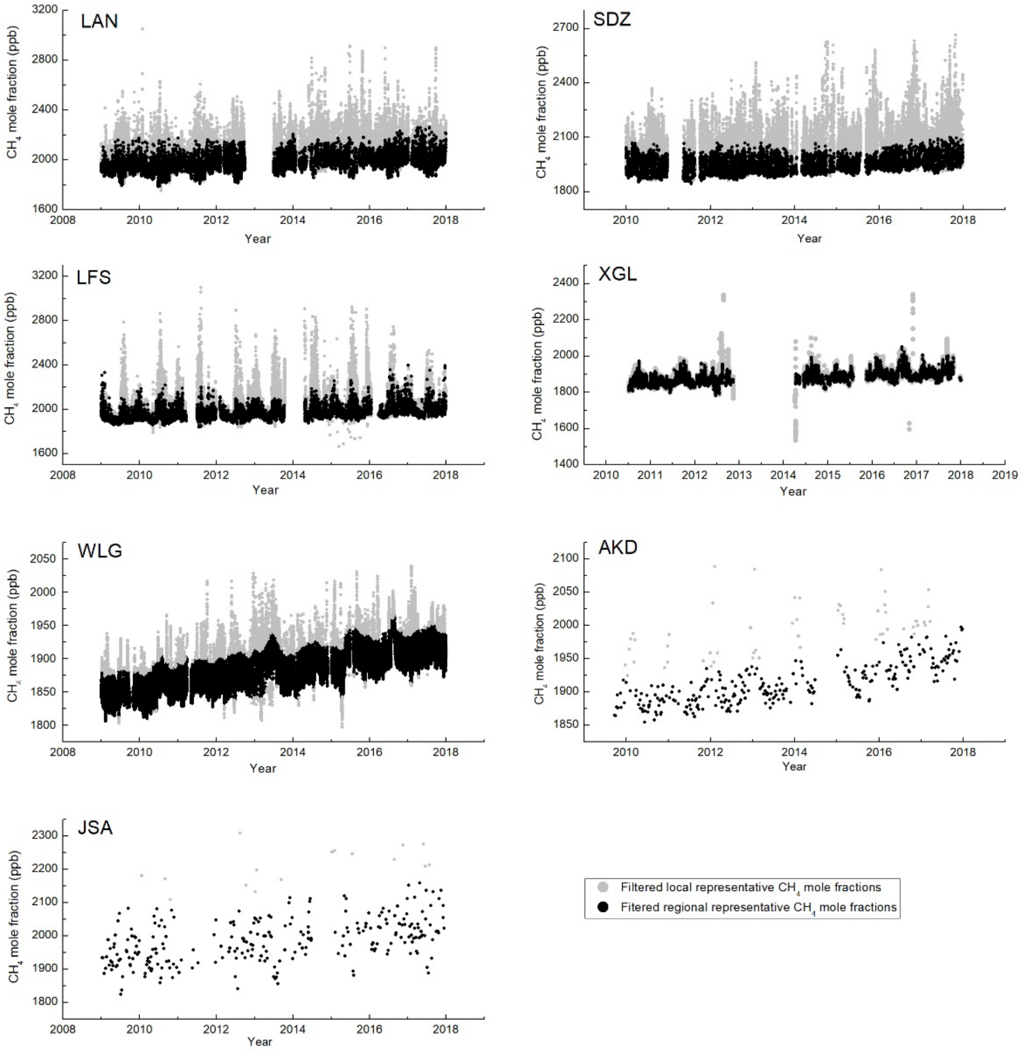

Figure S11. Hourly or weekly CH_4 mole fraction observations (ppb) over the Chinese sites.

References

1 Fang, S.-X., Zhou, L.-X., Masarie, K. A., Xu, L. & Rella, C. W. Study of atmospheric CH4 mole fractions at three WMO/GAW stations in China. *Journal of Geophysical Research: Atmospheres* **118**, 4874-4886, doi:https://doi.org/10.1002/jgrd.50284 (2013).

2 Wang, J. *et al.* Large Chinese land carbon sink estimated from atmospheric carbon dioxide data. *Nature* **586**, 720-723, doi:10.1038/s41586-020-2849-9 (2020).

3 Dlugokencky, E. J. *et al.* Conversion of NOAA atmospheric dry air CH4 mole fractions to a gravimetrically prepared standard scale. *Journal of Geophysical Research: Atmospheres* **110**, doi:https://doi.org/10.1029/2005JD006035 (2005).

4 Lang, P. M., Steele, L. P., Martin, R. C. & Masarie, K. A. Atmospheric methane data for the period 1983-1985 from the NOAA/GMCC global cooperative flask sampling network. NOAA Technical Memorandum ERL CMDL-1. (NOAA Climate Monitoring and Diagnostics Laboratory, Boulder, Colorado, USA, 1990).

5 Liu, C., Lu, M., Cui, J., Li, B. & Fang, C. Effects of straw carbon input on carbon dynamics in agricultural soils: a meta-analysis. *Global Change Biology* **20**, 1366-1381, doi:https://doi.org/10.1111/gcb.12517 (2014).

6 Hou, P. *et al.* Methane emissions from rice fields under continuous straw return in the middle-lower reaches of the Yangtze River. *Journal of Environmental Sciences* **25**, 1874-1881, doi:https://doi.org/10.1016/S1001-0742(12)60273-3 (2013).

7 Jiang, Y. *et al.* Acclimation of methane emissions from rice paddy fields to straw addition. *Science Advances* **5**, eaau9038, doi:10.1126/sciadv.aau9038 (2019).

8 Conrad, R. Microbial Ecology of Methanogens and Methanotrophs, in *Advances in Agronomy* Vol. 96, 1-63, Academic Press, doi: https://doi.org/10.1016/S0065-2113(07)96005-8 (2007).

9 Shi, Z. *et al.* Utilization characteristics, technical model and development suggestion on crop straw in China. *Journal of Agricultural Science and Technology* **21**, 8-16, doi:10.13304/j.nykjdb.2018.0314 (2019).

10 Yan, X., Cai, Z., Ohara, T. & Akimoto, H. Methane emission from rice fields in mainland China: Amount and seasonal and spatial distribution. *Journal of Geophysical Research: Atmospheres* **108**, doi:https://doi.org/10.1029/2002JD003182 (2003).

11 Zhang, G. *et al.* Residue usage and farmers′ recognition and attitude toward residue retention in China's croplands. *Journal of Agro-Environment Science* **36**, 981-988, doi:10.11654/jaes.2016-1505 (2017).

12 Cao, L. *et al.* China's aquaculture and the world's wild fisheries. *Science* **347**, 133-135, doi:10.1126/science.1260149 (2015).

13 Yuan, J. *et al.* Rapid growth in greenhouse gas emissions from the adoption of industrial-scale aquaculture. *Nature Climate Change* **9**, 318-322, doi:10.1038/s41558-019-0425-9

(2019).

14 Heald, C. L. *et al.* Comparative inverse analysis of satellite (MOPITT) and aircraft (TRACE-P) observations to estimate Asian sources of carbon monoxide. *Journal of Geophysical Research: Atmospheres* **109**, n/a-n/a, doi:10.1029/2004JD005185 (2004).

15 Maasakkers, J. D. *et al.* Global distribution of methane emissions, emission trends, and OH concentrations and trends inferred from an inversion of GOSAT satellite data for 2010–2015. *Atmos. Chem. Phys.* **19**, 7859-7881, doi:10.5194/acp-19-7859-2019 (2019).

16 Zhang, Y. *et al.* Monitoring global tropospheric OH concentrations using satellite observations of atmospheric methane. *Atmos. Chem. Phys.* **18**, 15959-15973, doi:10.5194/acp-18-15959-2018 (2018).

17 Lu, X. *et al.* Global methane budget and trend, 2010–2017: complementarity of inverse analyses using in situ (GLOBALVIEWplus CH4 ObsPack) and satellite (GOSAT) observations. *Atmos. Chem. Phys.* **21**, 4637-4657, doi:10.5194/acp-21-4637-2021 (2021).

18 Hansen, P. C. in *Computational Inverse Problems in Electrocardiology Advances in Computational Bioengineering* (ed P. Johnston) 119-142 (WIT Press, 2000).

19 Maasakkers, J. D. *et al.* 2010–2015 North American methane emissions, sectoral contributions, and trends: a high-resolution inversion of GOSAT observations of atmospheric methane. *Atmos. Chem. Phys.* **21**, 4339-4356, doi:10.5194/acp-21-4339-2021 (2021).

20 Lu, X. *et al.* Methane emissions in the United States, Canada, and Mexico: Evaluation of national methane emission inventories and sectoral trends by inverse analysis of in situ (GLOBALVIEWplus CH4 ObsPack) and satellite (GOSAT) atmospheric observations. *Atmos. Chem. Phys. Discuss.* **2021**, 1-41, doi:10.5194/acp-2021-671 (2021).

21 Sheng, J. *et al.* Sustained methane emissions from China after 2012 despite declining coal production and rice-cultivated area. *Environmental Research Letters* **16**, 104018, doi:10.1088/1748-9326/ac24d1 (2021).

22 Bergamaschi, P. et al., Atmospheric CH4 in the first decade of the 21st century: Inverse modeling analysis using SCIAMACHY satellite retrievals and NOAA surface measurements. *Journal of Geophysical Research: Atmospheres* **118**, 7350-7369, 10.1002/jgrd.50480 (2013).

23 Thompson, R. L. *et al.* Methane emissions in East Asia for 2000–2011 estimated using an atmospheric Bayesian inversion. *Journal of Geophysical Research: Atmospheres* **120**, 4352-4369, doi:10.1002/2014jd022394 (2015).

24 Miller, S. M. *et al.* China's coal mine methane regulations have not curbed growing emissions. *Nature Communications* **10**, 303, doi:10.1038/s41467-018-07891-7 (2019).

25 Wang, F. *et al.* Methane Emission Estimates by the Global High-Resolution Inverse Model Using National Inventories. *Remote Sensing* **11**, 2489, doi: 10.3390/rs11212489 (2019).

26 Janardanan, R. *et al.* Country-Scale Analysis of Methane Emissions with a High-Resolution

Inverse Model Using GOSAT and Surface Observations. *Remote Sensing* **12**, 375, doi:10.3390/rs11212489 (2020).

27 Saunois, M. *et al.* The Global Methane Budget 2000–2017. *Earth Syst. Sci. Data* **12**, 1561-1623, doi:10.5194/essd-12-1561-2020 (2020).

28 Qu, Z. et al., Global distribution of methane emissions: a comparative inverse analysis of observations from the TROPOMI and GOSAT satellite instruments. *Atmos. Chem. Phys.* **21**, 14159-14175, doi:10.5194/acp-21-14159-2021 (2021).

29 Wang, F. *et al.* Interannual variability on methane emissions in monsoon Asia derived from GOSAT and surface observations. *Environmental Research Letters* **16**, 024040, doi:10.1088/1748-9326/abd352 (2021).

30 Yin, Y. *et al.* Accelerating methane growth rate from 2010 to 2017: leading contributions from the tropics and East Asia. *Atmos. Chem. Phys.* **21**, 12631-12647, doi:10.5194/acp-21-12631-2021 (2021).

31 Zhang, Y. *et al.* Attribution of the accelerating increase in atmospheric methane during 2010–2018 by inverse analysis of GOSAT observations. *Atmos. Chem. Phys.* **21**, 3643-3666, doi:10.5194/acp-21-3643-2021 (2021).

32 Stavert, A.R. et al., Regional trends and drivers of the global methane budget. *Global Change Biology* **28**, 182-200, doi:10.1111/gcb.15901 (2022).

33 China Agriculture Yearbook 2010-2018. China Agriculture Press, Beijing (2018).

34 China Fishery Statistical Yearbook 2010-2018. China Agriculture Press, Beijing (2018).
